# Preclinical Study of ZSP1273, a Potent Antiviral Inhibitor of Cap Binding to the PB2 Subunit of Influenza A Polymerase

**DOI:** 10.3390/ph16030365

**Published:** 2023-02-27

**Authors:** Xiaoxin Chen, Qinhai Ma, Manyu Zhao, Yuqin Yao, Qianru Zhang, Miao Liu, Zifeng Yang, Wenbin Deng

**Affiliations:** 1 School of Pharmaceutical Science (Shenzhen), Sun Yat-Sen University, Shenzhen 518107, China; 2 State Key Laboratory of Respiratory Disease, National Clinical Research Center for Respiratory Disease, Guangzhou Institute of Respiratory Health, the First Affiliated Hospital of Guangzhou Medical University, Guangzhou 510030, China; 3 Molecular Toxicology Laboratory of Sichuan Provincial Education Office, Institute of Systems Epidemiology, West China School of Public Health and West China Fourth Hospital, Sichuan University, Chengdu 610041, China; 4 State Key Laboratory of Biotherapy and Cancer Center, West China Hospital, Sichuan University, Chengdu 610041, China

**Keywords:** influenza A, influenza A virus, RNA polymerase, ZSP1273, antiviral drug

## Abstract

The influenza A virus is highly contagious and often causes global pandemics. The prevalence of strains of the influenza A virus that are resistant to approved drugs is a huge challenge for the current clinical treatment of influenza A. RNA polymerase is a pivotal enzyme in the replication of the influenza A virus, and it is a promising target for anti-influenza A therapies. In this paper, we report a novel and potent anti-influenza-A-virus inhibitor, ZSP1273, targeting the influenza A virus RNA polymerase, especially for multidrug-resistant strains. The inhibitory activity of ZSP1273 on RNA polymerase activity was 0.562 ± 0.116 nM (IC_50_ value), which was better than that of the clinical candidate compound VX-787 with the same target. In vitro, the EC_50_ values of ZSP1273 on normal influenza A virus strains (i.e., H1N1 and H3N2) varied from 0.01 nM to 0.063 nM, which were better than those of the licensed drug oseltamivir. Moreover, oseltamivir-resistant strains, baloxavir-resistant strains, and highly pathogenic avian influenza strains were also sensitive to ZSP1273. In vivo, ZSP1273 effectively reduced influenza A virus titers in a dose-dependent manner in a murine model and maintained a high survival rate in mice. In addition, the inhibitory activity of ZSP1273 on influenza A virus infection was also observed in a ferret model. Pharmacokinetic studies showed the favorable pharmacokinetic characteristics of ZSP1273 in mice, rats, and beagle dogs after single-dose and continuous multiple-dose administration. In conclusion, ZSP1273 is a highly effective anti-influenza A virus replication inhibitor, especially against multidrug-resistant strains. ZSP1273 is currently being studied in phase III clinical trials.

## 1. Introduction

The influenza A virus, which can infect many species, is the main type of influenza virus that leads to outbreaks and global pandemics [1,2]. This virus can be further classified according to the glycoproteins on its envelope surface: hemagglutinin (HA) and neuraminidase (NA). Hemagglutinin has 18 different molecules (H1–H18), while neuraminidase has 11 molecules (N1–N11) [3]. At present, it has been confirmed that the serotypes that can infect humans include H1N1, H2N2, H3N2, H5N1, H1N2, H7N2, H7N3, H7N7, H7N9, H9N2, and H10N7, among which H1N1 and H3N2 are the main influenza A viruses that are routinely transmitted in humans [4,5].

**Figure 1:**
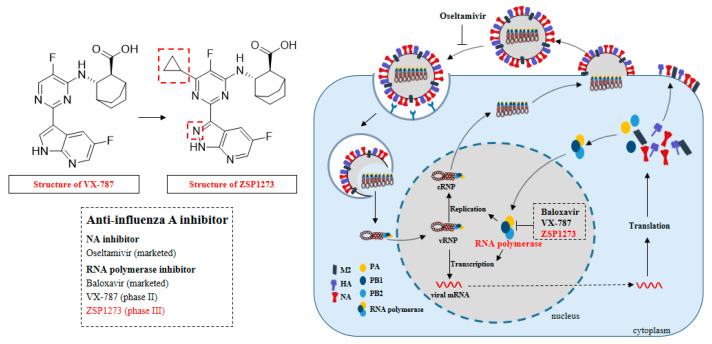
Therapeutic targets and small-molecule-targeted drugs for treatment of the influenza A virus.

The mechanism by which the influenza A virus causes influenza A depends on its replication in host cells. Generally, HA attaches the virus to the cell surface by binding to sialic acid and facilitates the virus’s entry into the cell to release genetic material [6]. The genome of the influenza A virus entering the host cell contains a total of eight RNA gene segments, of which the influenza A virus RNA polymerase that regulates the RNA synthesis of the virus is encoded by the first three sequences [3,7]. The influenza A virus RNA polymerase is a complex composed of polymerase basic protein 1 (PB1), polymerase basic protein 2 (PB2), and polymerase acidic protein (PA), which binds to the viral promoter [4]. The PB2 subunit is an essential part of the RNA polymerase, recognizing and “snatching” the 5′-capped primer of the host cell’s pre-mRNA, which is subsequently cleaved by PA as a primer for viral RNA synthesis, and then the viral RNA is used as a template to initiate viral RNA synthesis under the action of PB1 [8]. RNA fragments 4, 5, and 6 encode the viral glycoprotein HA, the viral nucleoprotein, and NA, respectively. RNA fragment 7 encodes a matrix protein (M1) and a membrane protein (M2). The M1 protein is involved in regulating the transport of viral RNA fragments in cells, while the M2 protein is a proton ion channel necessary for viral entry and exit [9]. The NS1 protein encoded by RNA fragment eight is a virulence factor that inhibits host antiviral responses [3]. Viral replication leads to the death of host cells, and NA can inhibit the binding of HA of new virus particles to sialic acid on the membranes of infected host cells to promote viral transmission [10] (Figure 1).

Anti-influenza drugs play a vital role in the prevention and treatment of influenza A. There are three kinds of anti-influenza drugs for treatment: NA inhibitors such as oseltamivir; RNA polymerase inhibitors such as baloxavir (mainly targeting PA); and M2-type ion channel blockers that inhibit the virus’s release of nucleic acids to host cells, such as amantadine and rimantadine [11,12]. However, amantadine and rimantadine are no longer recommended for the prevention and treatment of influenza A due to its high drug resistance [13]. Currently, the approved anti-influenza-A drugs—such as the NA inhibitor oseltamivir and the RNA polymerase inhibitor baloxavir—have achieved significant efficacy in clinical anti-influenza-A treatment. However, the emergence of oseltamivir- and baloxavir-resistant strains has made them less effective, especially in children and immunocompromised individuals with higher viral loads [14,15,16]. In 2007, the oseltamivir-resistant influenza A (H1N1) virus caused a global pandemic. In 2010–2011, an increasing number of oseltamivir-resistant cases were recognized [17]. Resistance to oseltamivir has also been reported in influenza A (H3N2) viruses, but less frequently than in influenza A (H1N1) viruses [3]. In addition, reduced susceptibility of the A/WSN/33(H1N1) virus to baloxavir has been reported [18]. Therefore, it is urgent to develop drugs that can effectively inhibit influenza A virus infection, especially for oseltamivir- and baloxavir-resistant virus strains (Figure 1).

ZSP1273 is a novel anti-influenza-A drug that targets RNA polymerase, which can effectively inhibit influenza A virus replication. The introduction of cypropyl into position six of VX-787′s molecular pyrimidine ring can cleverly occupy the hydrophobic binding pocket, greatly improving the antiviral activity of the compound. Meanwhile, in order to further balance the physical and chemical properties of the compound, ZSP1273 can be obtained by replacing azazindole with azazindazole (Figure 1). The purpose of this study was to explore the antiviral activity of ZSP1273 using in vitro and in vivo models—especially against oseltamivir- and baloxavir-resistant virus strains—and to evaluate the safety and pharmacokinetics of ZSP1273.

## 2. Results

### 2.1. Inhibitory Activity of ZSP1273 on Influenza A Virus RNA Polymerase

RNA polymerase is an important enzyme in the replication of the influenza A virus. In order to analyze the inhibitory effect of ZSP1273 on the influenza virus polymerase, the influenza virus polymerase reporter plasmid system was constructed in vitro, transfected into 293T cells, and the test compound was added. Later, the expression level of polymerase was compared with that of a control group and a drug-free polymerase control group without the drug polymerase. Moreover, the % inhibition of the drug on the influenza virus polymerase was calculated to determine the IC_50_ value. The RNA polymerase inhibitor VX-787 and favipiravir were used as control compounds. The results showed that ZSP1273 significantly inhibited influenza virus polymerase, with a corresponding IC_50_ value of 0.562 ± 0.116 nM, which was better than those of VX-787 (1.449 ± 0.34 nM) and favipiravir (34,087.67 ± 16,009.21 nM) (see Table A1 for details).

### 2.2. Kinase Inhibition Assay of ZSP1273 against Human Kinases

Antiviral drugs may have side effects if they bind to human kinases. We therefore explored the effects of ZSP1273 on host kinase binding. The pan-kinase inhibitor staurosporine was used as a positive control. Kinase activity was measured using Z’-LYTE^®^ technology (Invitrogen, Waltham, MA, USA) based on the principle of fluorescence resonance energy transfer (FRET). The IC_50_ value was calculated by determining the % inhibition of the kinase by the drug; the results showed that ZSP1273 demonstrated no inhibitory activity against other kinases (with IC_50_ values more than 10 μM), while the IC_50_ of VX-787 ranged from 1000 nM to 10,000 nM against GSK3 beta, MAP4K4, MINK, and TNIK, but no significant inhibition against other kinases by VX-787 was observed (Table A2). Staurosporine exhibited inhibitory activity against most kinases.

### 2.3. Inhibitory Activity of ZSP1273 against Human GPCRs

G-protein-coupled receptors (GPCRs) are involved in various signal transduction pathways and regulate many physiological processes in human cells [19]. To evaluate the agonistic or antagonistic effects of ZSP1273 on human normal GPCRs at the cellular level, the corresponding GPCR targets were stably expressed in host cells (CHO or HEK293), and the agonistic or antagonistic effects of the test compounds against the corresponding targets were detected based on the change in the abundance of calcium ions in the subsequent signaling pathway. Fluorescence imaging plate reader (FLIPR TM) technology is an important method to detect changes in intracellular calcium ion flux. In this study, the agonistic or antagonistic effects of the test compounds against the targets were tested using FLIPR technology at a cellular level, and their safety was preliminarily evaluated. As shown in Table A3 and Table A4, compared with the EC_50_ values of the positive control, ZSP1273 demonstrated no agonistic activity (EC_50_ > the maximum use concentration of 12 μM) against all 22 GPCR targets tested. In the GPCR antagonist screening assay, compared with the IC_50_ values of the positive control, ZSP1273 also showed no activity (IC_50_ > the maximum use concentration of 10 μM) against any of the 22 GPCR targets. These results indicate that ZSP1273 exerts no obvious toxicity against GPCRs in the host.

### 2.4. Inhibitory Activity of ZSP1273 on the hERG Potassium Channel

**Figure 2:**
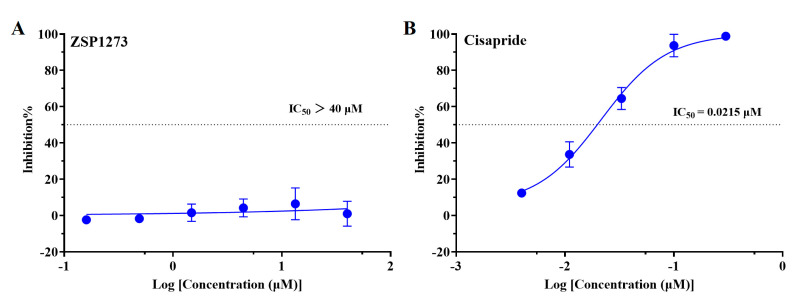
(**A**) Dose−response curves and IC_50_ values of ZSP1273 on the hERG potassium channel current; (**B**) Dose−response curves and IC_50_ values of cisapride on the hERG potassium channel current.The IC_50_ values of the compounds were calculated by fitting them with the following equation: I/Io = 1/{1 + ([C]/IC_50_) ^ n}, where Io and I are the hERG potassium current amplitudes before and after administration, respectively, while [C] is the compound concentration, and n is the Hill factor.

The human ether-a-go-go-related gene (hERG) potassium channel is important in regulating cardiac excitability [20]. To evaluate whether ZSP1273 affects the function of the host’s heart, the inhibitory effect of ZSP1273 on the hERG potassium channel was evaluated. When testing CHO (Chinese Hamster Ovary) cells with stable expression of the hERG potassium channel, the hERG potassium channel current was recorded at room temperature using the whole-cell patch-clamp technique. The IC_50_ value was calculated based on the % inhibition of the compound on the hERG potassium current. In this assay, the IC_50_ value of the positive control compound cisapride was 21.5 nM, indicating that the test system was stable and reliable. On the CHO cells with stable expression of the hERG potassium channel, the inhibition percentage of ZSP1273 at 40 µM on the hERG potassium current was less than 50% at 1-min administration. The inhibition percentages of compounds at different concentrations regarding the hERG potassium channel current, along with the corresponding dose–response curves and IC_50_ values, are shown in Figure 2. These results indicate that ZSP1273 demonstrated no obvious toxicity toward the potassium channel of the host’s heart.

### 2.5. In Vitro Antiviral Activity of ZSP1273 on H1N1 and H3N2

Currently, H1N1 and H3N2 are the main influenza A viruses that are most frequently transmitted among humans, often causing outbreaks or pandemics. The cytopathic effect (CPE) method was used to test the in vitro antiviral activity of ZSP1273 against the H1N1 and H3N2 strains. The ability of compounds to inhibit viral-induced cytopathic effects caused by different influenza virus strains was assessed by monitoring cell viability in MDCK cells. The cytotoxicity of the compounds was assessed in parallel under the same conditions, but without viral infection. The inhibitory activity and cell viability of the compounds were analyzed by nonlinear fitting using GraphPad Prism (Version 5) software to calculate the median effective concentration (EC_50_) and half-maximal cytotoxic concentration (CC_50_) of the compounds. The RNA polymerase inhibitor baloxavir (marketed), VX-787, and the NA inhibitor oseltamivir (marketed) were used as positive controls. As shown in Table 1, ZSP1273 exhibited high inhibitory activity against all six tested H1N1 and H3N2 influenza A virus strains, with an average EC_50_ value range of 0.012–0.063 nM. Its inhibitory activity was higher than that of VX-787 and oseltamivir. Subsequently, the effects of ZSP1273 on the influenza A/California/07/2009 (H1N1) pdm09 and A/California/2/2014 (H3N2) strains, which have caused epidemics in the United States in recent years, were evaluated. The results also showed that the EC_50_ values of ZSP1273 against the two influenza viruses were 0.02 ± 0.003 nM and 0.01 ± 0.0002 nM, respectively (Table A5). The cytotoxicity test was performed in parallel on MDCK cells, showing that ZSP1273 exhibited toxicity within the detectable concentration range, and the mean CC_50_ values were 1.632 µM and 1.777 µM under 33 °C and 37 °C culture conditions, respectively (Table 1). ZSP1273, compared with its in vitro activity against IFVA, exhibited good cytotoxic selectivity (SI > 25,000-fold).

### 2.6. In Vitro Antiviral Activity of ZSP1273 against Oseltamivir-Resistant Strains

The antiviral activity of ZSP1273 against oseltamivir-resistant influenza virus strain A/Weiss/43 (H1N1) was detected by the CPE method in MDCK cells. In comparison to oseltamivir, ZSP1273 also demonstrated high inhibitory activity against all drug-resistant strains of influenza A virus, with mean EC_50_ values of 0.014 nM and 0.017 nM, respectively, which are similar to the EC_50_ value against wild-type influenza A/PR/8/34 (H1N1) (with EC_50_ values of 0.042 nM). For the same target inhibitor group, the antiviral activity of VX-787 against oseltamivir-resistant strains was lower than that of ZSP1273, with EC_50_ values of 0.234 nM and 0.216 nM, respectively (Table 2). The mean CC_50_ values of blank MDCK cells without viral transfection were 1.632 µM and 1.777 µM at 33 °C and 37 °C, respectively. The results showed that ZSP1273 exhibited good cytotoxic selectivity.

### 2.7. In Vitro Antiviral Activity of ZSP1273 against Baloxavir-Resistant Influenza Virus

The PI38T site mutation of the influenza A/PR/8/34 (H1N1) strain caused by baloxavir reduced the sensitivity of the strain to baloxavir [21]. The antiviral activity of ZSP1273 against baloxavir-resistant influenza A/PR/8/34 (H1N1) was then evaluated by the CPE method in MDCK cells. Baloxavir and VX-787 were used as reference compounds. ZSP1273 showed an EC_50_ value of 0.028 nM against baloxavir-resistant influenza A/PR/8/34 (H1N1) (Table 2), which is similar to its EC_50_ value against wild-type influenza A/PR/8/34 (H1N1) (with an EC_50_ value of 0.042 nM).

### 2.8. In Vitro Antiviral Activity of ZSP1273 against Highly Pathogenic Avian Influenza Viruses (H7N9 and H5N6)

Highly pathogenic avian influenza viruses may also be transmitted to humans and cause outbreaks. The antiviral activity of ZSP1273 against avian influenza virus A/Anhui/01/2013 (H7N9) was then determined by the CPE method in MDCK cells. The 50% inhibitory concentration (IC_50_) was calculated by the Reed–Muench method. Compared with VX-787 and oseltamivir, ZSP1273 had stronger inhibitory effects on avian influenza viruses A/Anhui/01/2013 (H7N9), A/Qingyuan/GIRD01/2017 (H7N9), and A/Guangzhou/39715/2014 (H5N6) in vitro, with the IC_50_ values of 0.627 ± 0.312 nM, 0.777 ± 0.427 nM, and 0.245 ± 0.03 nM, respectively (Table 2), which were weaker than the EC_50_ values against influenza A virus strains H1N1 and H3N2 (with EC_50_ values ranging from ~0.012 to 0.063 nM).

### 2.9. Combination Experiments of ZSP1273 with Oseltamivir In Vitro

**Figure 3:**
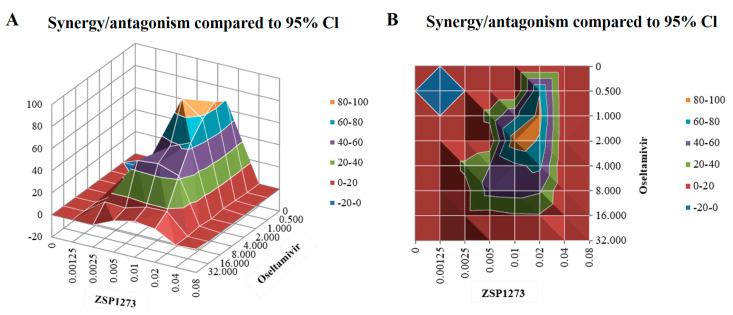
Efficacy of ZSP1273 in combination with oseltamivir in vitro: (**A**) Positive and negative synergy diagrams of the combined administration. A positive combined index indicates a synergistic effect, while a negative combined index indicates an antagonistic effect. If the absolute value of the index is <25, it indicates an additive effect; if the above value is in the range of 25–50, it indicates a mild but clear synergistic or antagonistic effect; if the above value is in the range of 50–100, it indicates a moderate synergistic or antagonistic effect, which is possibly important for in vivo effects; if the above value is >100, it indicates a high degree of synergistic or antagonistic effect, which is probably important for in vivo effects. (**B**) An aerial view of (**A**).

The combined effects of ZSP1273 and oseltamivir in vitro were evaluated by an influenza virus cytopathic assay. The viability of cells in each well was measured with CCK-8. The assay data were processed using the MacSynergy software, and the efficacy parameters of ZSP1273 in combination with oseltamivir were analyzed. The results showed that the synergy and antagonism indices of ZSP1273 combined with oseltamivir were 852.41 and −0.19, respectively, suggesting that the two have strong synergistic effects (Figure 3A,B). These results support the combined application of ZSP1273 and oseltamivir in vitro.

### 2.10. Evaluation of the Anti-Influenza Virus Specificity of ZSP1273

To further evaluate the specificity of the inhibitory activity of ZSP1273 against the influenza A virus, the inhibitory activity of ZSP1273 against seven other viruses and its effects on the proliferation of corresponding infected cells were evaluated. The viruses included positive-strand RNA viruses (human rhinovirus (HRV), human enterovirus 71 (EV71), and hepatitis C virus (HCV)), negative-strand RNA viruses (respiratory syncytial virus (RSV) and human parainfluenza virus type 3 (HPIV-3)), a DNA virus (herpes simplex virus type 1 (HSV-1)), and a hepatotropic virus (hepatitis B virus (HBV)). Among the tested viruses, the five virus assays were carried out using the cytopathic effect (CPE) method induced by viral infection to determine the antiviral activity of WXFL20100273. The hepatitis B virus assay was performed using the quantitative PCR (qPCR) method on the HepG2.2.15 cell line, with stable transfection of HBV expression. The hepatitis C virus assay was performed using hepatitis C virus genotype 1b replicon cells. The results showed that ZSP1273 exhibited no inhibitory activity against the other six viruses (EC_50_ > the maximum concentration of 10 µM) but had some inhibitory effect on HRV-1B (with an EC_50_ of 7.88 µM). In addition, ZSP1273 showed no significant cytotoxicity to the seven cell lines in the detectable concentration range (CC_50_ > the maximum concentration) (Table 3).

### 2.11. Efficacy Experiments with ZSP1273 in a Murine Infection Model

#### 2.11.1. Murine Infection Experiment I

**Figure 4:**
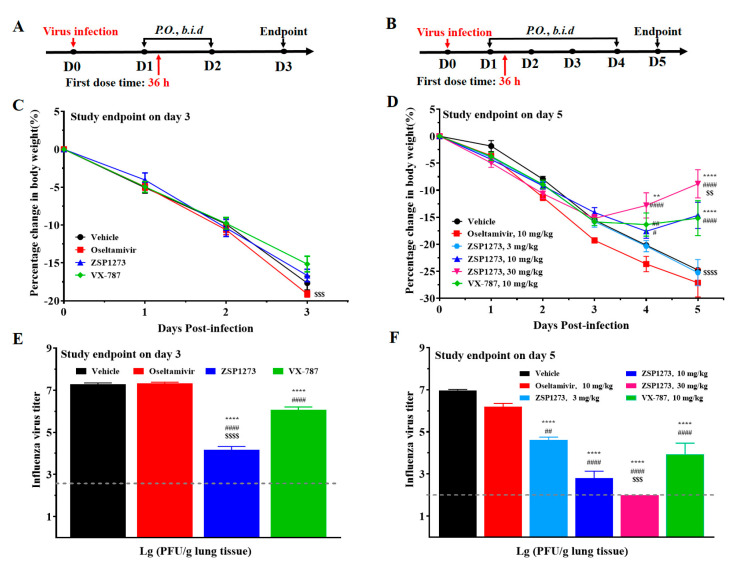
The antiviral activity of ZSP1273 in murine infection experiment I (n = 6, mean ± SEM): (**A**) Flowchart of the experiment (study endpoint on day 3); the day of H1N1 infection is defined as day 0, the first day after infection is denoted as day 1, and so forth. (**B**) Flowchart of the experiment (study endpoint on day 5). (**C**) Change in body weight on day 3. (**D**) Body weight change on day 5. (**E**) Influenza A virus titers on day 3. (**F**) Influenza A virus titers on day 5. The viral titer lower than the LOD (2.0 log10) was calculated as 2.0. Note: ** *p* < 0.01, **** *p* < 0.0001 (vs. vehicle); # *p* < 0.05, ## *p* < 0.01, #### *p* < 0.0001 (vs. oseltamivir); $$ *p* < 0.01, $$$ *p* < 0.001, $$$$ *p* < 0.0001 (vs. VX−787).

In order to analyze the effects of ZSP1273 on influenza A virus replication at 36 h after infection based on the influenza virus titers in lung tissues, a H1N1-infected murine model (WSN/33, inoculation size of 1.0 × 10^4^ PFU/mouse) was established. Oseltamivir and VX-787 were used as reference compounds. The flowcharts of the experiments are shown in Figure 4A,B, and changes in the mice’s weight after infection are shown in Figure 4C,D. All mice began to lose body weight on day 1 post-infection, whereas mice in the ZSP1273 (30 mg/kg) and VX-787 (10 mg/kg) groups began to regain body weight on day 4, and mice in the ZSP1273 (10 mg/kg) group began to regain body weight on day 5.

On days 3 and 5, the lung tissues of mice were collected for viral plaque assays to measure their viral titers. For groups with the study endpoint on day 3, compared with the vehicle group, both VX-787 (10 mg/kg) and ZSP1273 (10 mg/kg) significantly reduced the viral titers in murine lung tissues, with the viral load decreasing by 1.2 log10 and 3.1 log10, respectively (Figure 4E). However, there was no significant decrease in viral titers in the oseltamivir (10 mg/kg) group.

For groups with the study endpoint on day 5, compared with the vehicle group, ZSP1273 (3, 10, and 30 mg/kg) significantly reduced viral titers in murine lung tissues in a dose-dependent manner, with the corresponding viral load decreases of 2.37 log10, 4.18 log10, and 4.97 log10, respectively (Figure 4F). In the 30 mg/kg ZSP1273 group, the viral titer was lower than the limit of detection (LOD), at 2.0 log10. The VX-787 (10 mg/kg) group also showed significant viral inhibition, with the viral titer in lung tissues reduced by 3.04 log10 (Figure 4F). The viral load in the oseltamivir (10 mg/kg) group decreased by 0.77 log10.

#### 2.11.2. Murine Infection Experiment II

**Figure 5:**
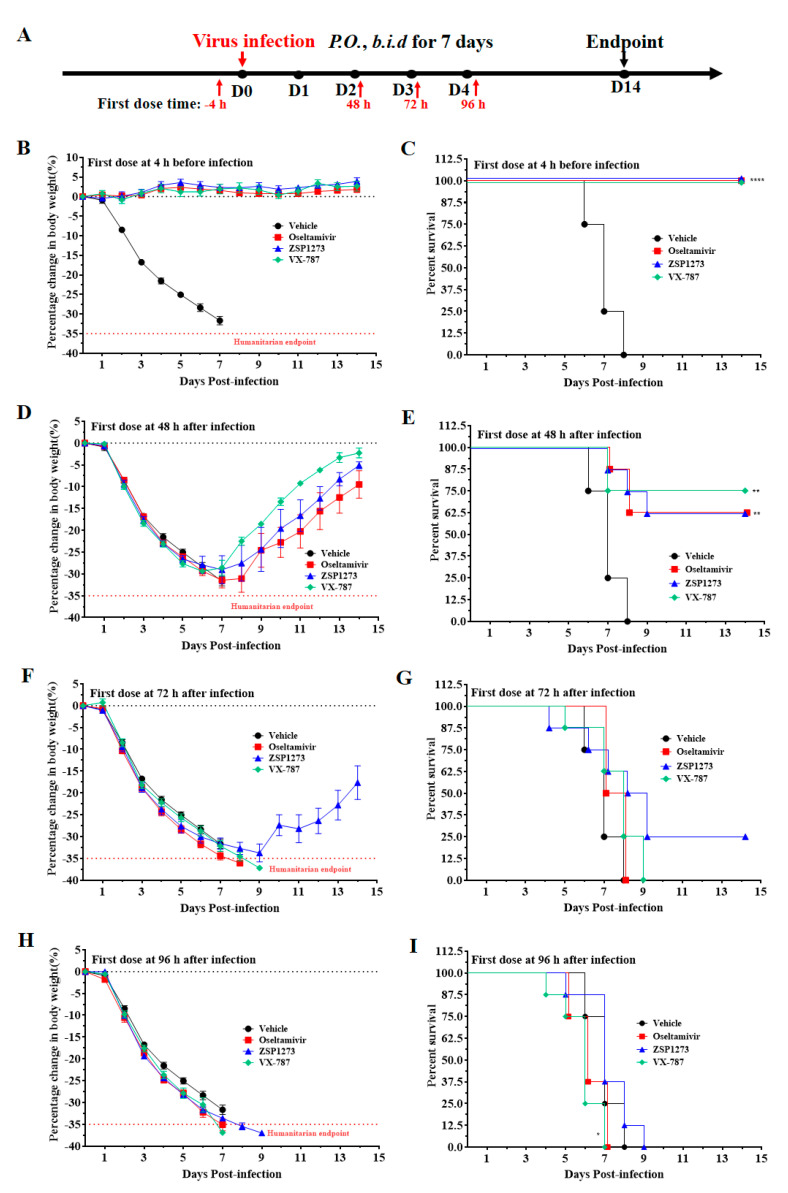
The antiviral activity of ZSP1273 in murine infection experiment II (n = 8, mean ± SEM): (**A**) Flowchart of the experiments: the day of H1N1 infection is defined as day 0, the first day after infection is denoted as day 1, and so forth. (**B**,**C**) Changes in the weight and survival rate, respectively, of mice in each group at 4 h after infection. (**D**,**E**) Changes in the weight and survival rate, respectively, of mice in each group at 48 h after infection. (**F**,**G**) Changes in the weight and survival rate, respectively, of mice in each group at 72 h after infection. (**H**,**I**) Changes in the weight and survival rate, respectively, of mice in each group at 96 h after infection. Based on the weight on the day of infection, a mouse with more than 35% weight loss (indicated by a red dashed line) would be considered dead, according to the IACUC. The survival analysis was performed by the Kaplan-Meier test; * *p* < 0.05, ** *p* < 0.01, **** *p* < 0.0001, compared with the vehicle group.

Based on the exploration of the antiviral effects of ZSP1273 administered at 36 h after infection in the previous experiments, a murine model of H1N1 infection (WSN/33, inoculation size of 1.0 × 10^4^ PFU/mouse) was used to evaluate the antiviral efficacy of 10 mg/kg ZSP1273 for preventive (at 4 h before infection) and therapeutic (at 48 h, 72 h, and 96 h after infection) treatments. The flowchart of the experiment is shown in Figure 5A. After the viral infection, the weight of mice in the vehicle group decreased continuously from the second day on, and all mice died on the eighth day. In the 4-h pre-infection administration test, ZSP1273, oseltamivir, and VX-787 did not significantly reduce the body weight of mice at the dose of 10 mg/kg compared with the vehicle group, and they maintained a 100% survival rate (Figure 5B,C). In the 48-h post-infection administration test, ZSP1273, oseltamivir, and VX-787 had no effect on the weight of mice at the dose of 10 mg/kg, but they could improve the survival rate of mice compared with the vehicle group (Figure 5D,E). In the 72-h post-infection administration test, the weight of mice in the oseltamivir and VX-787 groups continued to decrease; the body weight of the mice in the ZSP1273 group first decreased, reaching its minimum on the 9th day, and then the body weight of the surviving mice began to increase (Figure 5F). Ultimately, mice in the ZSP1273 group had a 25% survival rate, while all mice in the VX-787 and oseltamivir groups died (Figure 5G). All mice showed significant weight loss in the dosing test 96 h after the infection (Figure 5H). Mice in the oseltamivir and VX-787 groups died after 7 h—earlier than in the vehicle group—while mice in the ZSP1273 group died later than those in the vehicle group (Figure 5I).

#### 2.11.3. Murine Infection Experiment III

**Figure 6:**
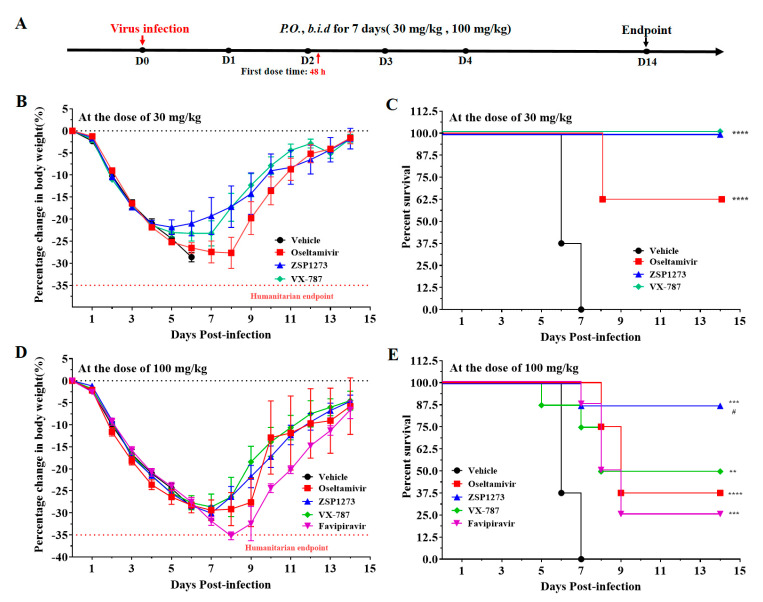
The antiviral activity of ZSP1273 in murine infection experiment III (n = 8, mean ± SEM): (**A**) Flowchart of the experiments: the day of H1N1 infection is defined as day 0, the first day after infection is denoted as day 1, and so forth. (**B**,**C**) Changes in the weight and survival rate, respectively, of mice in each group after infection at the dose of 30 mg/kg. (**D**,**E**) Changes in the weight and survival rate, respectively, of mice in each group after infection at the dose of 100 mg/kg. Based on the weight on the day of infection, a mouse with more than 35% weight loss (indicated by the dashed line) would be considered dead, according to the IACUC. Error bars represent the standard error of the mean value. Note: ** *p* < 0.01, *** *p* < 0.001, **** *p* < 0.0001 (vs. vehicle); # *p* < 0.05 (vs. favipiravir, 100 mg/kg).

Based on the previous experiments, a murine model of influenza A virus infection (WSN/33, inoculation size of 1.0 × 10^4^ PFU/mouse) was employed to analyze the antiviral efficacy of ZSP1273 administered at higher doses (30 mg/kg and 100 mg/kg) 48 h after infection by observing the weight loss and viability after infection. The antiviral efficacy was compared with that of the reference compounds, oseltamivir phosphate, VX-787, and favipiravir. The antiviral effect of ZSP1273 administered at higher doses (30 mg/kg and 100 mg/kg) was analyzed 48 h after infection using a murine model of influenza A virus infection. The flowchart of the experiment is shown in Figure 6A. In the 30 mg/kg dose group, the weight of mice in all groups began to decrease on day 1. The weight of surviving mice in the ZSP1273, VX-787, and oseltamivir groups increased gradually on the 6th^,^ 8th, and 9th days, respectively (Figure 6B). The survival rate of mice in the ZSP1273 and VX-787 groups was 100%—higher than that in the oseltamivir and vehicle groups (Figure 6C). In the 100 mg/kg dose groups, the body weight of the surviving mice in the ZSP1273 and VX-787 groups began to recover on day 8, and the mice that survived in the oseltamivir and favipiravir groups gradually recovered on day 9 (Figure 6D). At the dose of 30 mg/kg, no mice died in the ZSP1273 and VX-787 groups, but three mice died in the oseltamivir group. At the dose of 100 mg/kg, one, four, five, and six animals died in the ZSP1273, VX-787, oseltamivir, and favipiravir groups, respectively (Figure 6E).

#### 2.11.4. Murine Infection Experiment IV

**Figure 7:**
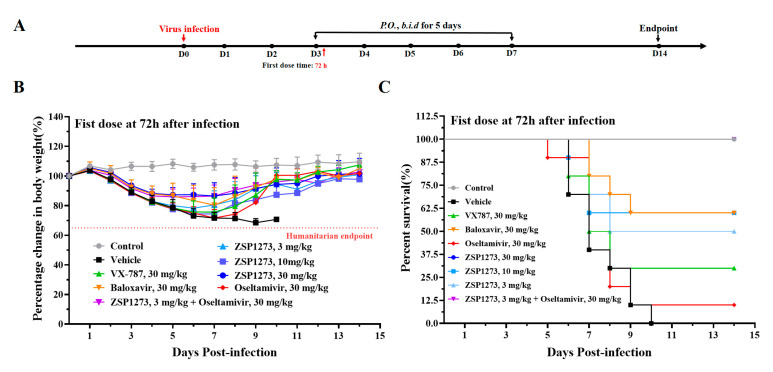
The antiviral activity of ZSP1273 in murine infection experiment IV (n = 10, mean ± SEM): (**A**) Flowchart of the experiments: the day of H1N1 infection is defined as day 0, the first day after infection is denoted as day 1, and so forth. (**B**,**C**) Changes in the weight and survival rate, respectively, of mice in each group after infection. Based on the weight on the day of infection, a mouse with more than 35% weight loss (indicated by the dashed line) would be considered dead, according to the IACUC.

To find out how the drug protects animals from death as a result of viral infection, a murine model of 2LD_50_ (lethal dose) influenza A virus infection (A/PR/8/34, inoculation size of 2.0 × 10^5^ PFU/mouse) was employed 72 h after infection by observing the weight loss and viability after infection. The antiviral efficacy was compared with that of the reference compounds, oseltamivir phosphate, VX-787, and baloxavir. It is important to note that we included a combination dosing group in this trial (ZSP1273 at 3 mg/kg + oseltamivir at 30 mg/kg). The flowchart of the experiment is shown in Figure 7A. After viral infection, the weight of mice in the vehicle group decreased continuously from the second day; the mice developed shortness of breath, crouching and trembling, weight loss, and other symptoms, and all mice died on the 11th day. The body weight of mice in each administration group tended to increase steadily (Figure 7B). After 14 days of continuous observation, the mice in the control group were in good condition, and no deaths were observed, while the mortality rate of the mice in the virus group was 100%. The results showed that the mortality rate of mice in the baloxavir group was 40%, the mortality rate of mice in the VX-787 group was 70%, and the mortality rate of mice in the oseltamivir group was 90%. No mice died in the ZSP1273 (30 mg/kg) group, and the mortality of mice in the ZSP1273 (10 mg/kg) group was 40%. The mortality rate was 50% in the ZSP1273 (3 mg/kg) group, while no mice died in the combination group (Figure 7C).

#### 2.11.5. Murine Infection Experiment V

**Figure 8:**
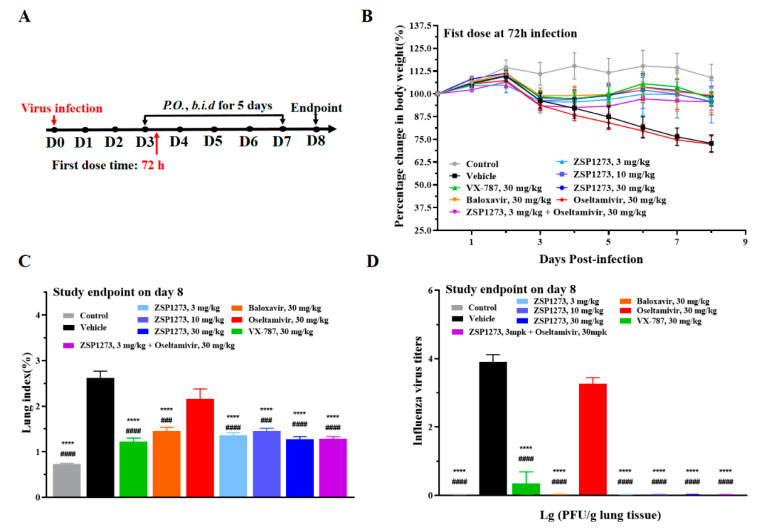
The antiviral activity of ZSP1273 in murine infection experiment V (n = 10, mean ± SEM): (**A**) Flowchart of the experiments: the day of H1N1 infection is defined as day 0, the first day after infection is denoted as day 1, and so forth. (**B**) Changes in the weight of mice in each group after infection. (**C**) Lung index—which is a ratio of the lung weight to body weight—on day 8 in each group. (**D**) Influenza A viral titers on day 8. Error bars represent the standard error of the mean value. Note: **** *p* < 0.0001 (vs. vehicle); ### *p* < 0.001, #### *p* < 0.0001 (vs. oseltamivir).

A murine model of LD_50_ (non-lethal dose) influenza A virus infection was established in order to analyze the effects of ZSP1273 and a combination dosing regimen on influenza A virus replication at 72 h after infection, based on the influenza virus titers in lung tissues. Oseltamivir, VX-787, and baloxavir were used as reference compounds. The flowchart of the experiments is shown in Figure 8A, and changes in the mice’s weight after infection are shown in Figure 8B. After infection with the influenza virus, the mice in the vehicle group lost weight continuously over time. Except for the oseltamivir group, the body weight of the mice in the other administration groups tended to change steadily.

On day 8, the lung tissues of mice were collected and weighed to calculate the lung index, which is a ratio of lung weight to body weight used to indicate the protective effect of a drug on the lungs and measure viral titers. The lung index of mice at all three doses of ZSP1273 was significantly decreased. At the same dose, the lung index of mice treated with ZSP1273 was lower than that of mice treated with baloxavir, but it was not significantly different from that of mice treated with VX-787. Importantly, compared with the oseltamivir (30 mg/kg) alone group, the lung index of the combined drug group decreased significantly (Figure 8C). On the 8th day after infection, the viral titers in the lung tissues of mice in the three dosage groups of ZSP1273 were all 0, indicating significant inhibition of the increase in viral titers in lung tissue caused by influenza virus infection, and the inhibition ability was better than that of VX-787. Compared with the oseltamivir (30 mg/kg) monotherapy group, the viral titer in the lung tissue of the combination group was significantly decreased (Figure 8D).

### 2.12. Efficacy Experiments with ZSP1273 in the Ferret Infection Model

**Figure 9:**
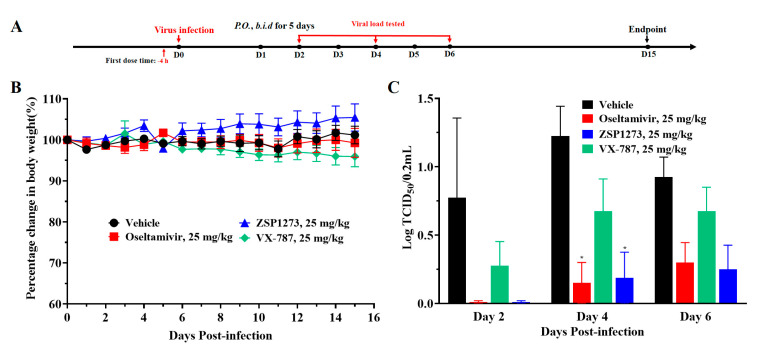
The antiviral activity of ZSP1273 in the ferret infection model (mean ± SEM): (**A**) Flowchart of the experiments: the day of H1N1 infection is defined as day 0, the first day after infection is denoted as day 1, and so forth. (**B**) Body weight changes of ferrets in each group with H1N1 infection after treatment. (**C**) Effects of oral administration of ZSP1273 on the viral load in nasal lavage fluid in the ferret influenza model. The statistical comparisons between each group and the vehicle group were performed by two-way ANOVA, with *p* < 0.05 indicating significant differences; * *p* < 0.05 (vs. vehicle); error bars represent the standard error of the mean value.

To further evaluate the inhibitory effect of ZSP1273 on influenza A virus infection in vivo, we established a ferret model of H1N1 infection (virus/California/07/2009, inoculation size of 1.0 × 10^5^ PFU/mouse); oseltamivir and VX-787 were used as reference compounds. The mean body weight of ferrets in all groups, except for the ZSP1273 group, decreased slightly within 2 days after infection. The flowchart of the experiment is shown in Figure 9A. The weight of the ferrets in the vehicle group returned to pre-infection levels and continued for more than 3 days after day 12 (Figure 9B). As shown in Figure 9C, compared with the vehicle group, the viral load in nasal lavage fluid decreased significantly in both the ZSP1273 group and the oseltamivir group on day 4 (*p* < 0.05), while the viral load in the VX-787 treatment group decreased but showed no statistical significance (*p* > 0.05). Meanwhile, on day 6, the viral load in all groups decreased but showed no statistical significance (*p* > 0.05).

### 2.13. Pharmacokinetic Study of Single- and Multi-Dose ZSP1273 Given by Oral or Intravenous Administration in Rats, Beagle Dogs, and Mice

**Figure 10:**
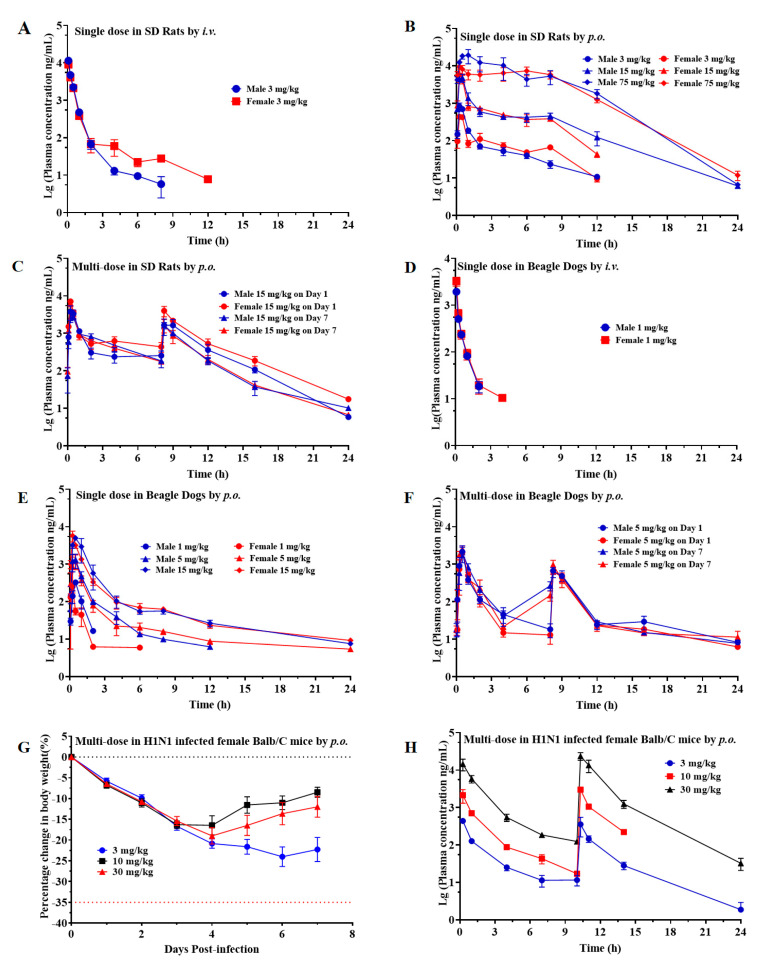
Pharmacokinetic study of ZSP1273 in SD rats, beagle dogs, and mice (n = 3, mean ± SEM): (**A**) The 24 h concentration–time curves of single-dose ZSP1273 in rats administered *i.v.* (**B**) The 24 h concentration-time curves of single-dose ZSP1273 in rats administered *p.o.* (**C**) The 24 h concentration-time curves of multi-dose ZSP1273 in rats administered *p.o.* (**D**) The 24 h concentration-time curves of single-dose ZSP1273 in rats administered *i.v.* (**E**) The 24 h concentration-time curves of single-dose ZSP1273 in beagle dogs administered *p.o.* (**F**) The 24 h concentration-time curves of multi-dose ZSP1273 in beagle dogs administered *p.o.* (**G**) Changes in the weight of H1N1-infected female BALB/c mice over 7 days. (**H**) The 24 h concentration-time curves of continuous multi-dose ZSP1273 (3, 10, and 30 mg/kg, twice daily, for 7 days) in H1N1-infected female BALB/c mice administered *p.o.* Error bars represent the standard error of the mean value.

The pharmacokinetic characteristics of ZSP1273 were analyzed in models using SD rats, beagle dogs, and mice. For the IV administration of ZSP1273 at 3 mg/kg in male and female SD rats, ZSP1273 showed a plasma clearance (CL) of 12.9 ± 2.23 mL/min/kg with a half-life (T_1/2_) of 2.11 ± 1.46 h. The volume of distribution (V_dss_) was 0.508 ± 0.241 L/kg, while the area under the plasma concentration–time curve from time zero to the last quantifiable concentration (AUC_0-last_) value was 3950 ± 773 ng·h/mL. Following oral administration of ZSP1273 at 3, 15, and 75 mg/kg in male and female SD rats, the AUC_0-last_ values of ZSP1273 were 899 ± 213, 7490 ± 1760, and 77,700 ± 38,800 ng·h/mL, respectively. The C_max_ values were 661 ± 304, 5620 ± 2610, and 17,200 ± 9100 ng/mL, respectively (Figure 10A). Following single oral administration of ZSP1273 at 3, 15, and 75 mg/kg in SD rats, the AUC_0-last_ values of ZSP1273 were 899 ± 213 ng·h/mL, 7490 ± 1760 ng·h/mL, and 77,700 ± 38,800 ng·h/mL, respectively, while the C_max_ values for each group were 661 ± 304 ng/mL, 5620 ± 2610 ng/mL, and 17,200 ± 9100 ng/mL, respectively. Moreover, there were no gender differences in the C_max_ and AUC_0-last_ values after oral administration of 3, 15, and 75 mg/kg ZSP1273 (Figure 10B). The mean oral bioavailability was 22.8% at 3 mg/kg. Subsequently, male and female SD rats were orally administered 15 mg/kg ZSP1273 twice daily for 7 consecutive days. The C_max_ and AUC_0-last_ values on day 1 were 5680 ± 2470 ng/mL and 12,100 ± 5110 ng·h/mL, respectively, while on day 7, they were 4810 ± 2150 ng/mL and 8390 ± 2110 ng·h/mL, respectively (Figure 10C). No significant accumulation of ZSP1273 was observed in SD rats after 7 days of dosing at the 15 mg/kg dose twice daily *p.o.* compared with day 1 (AUC_0-last_ and C_max_).

For the IV administration of ZSP1273 at 1 mg/kg in male and female beagle dogs, ZSP1273 showed a plasma clearance (CL) of 25.8 ± 7.60 mL/min/kg with a half-life (T_1/2_) of 0.457 ± 0.168 h. The volume of distribution (V_dss_) was 0.470 ± 0.0848 L/kg, while the area under the plasma concentration–time curve from time zero to the last quantifiable concentration (AUC_0-last_) value was 791 ± 329 h·ng/mL (Figure 10D). Following oral administration of ZSP1273 at 1, 5, and 15 mg/kg in male and female beagle dogs, the AUC_0-last_ values of ZSP1273 were 211 ± 62.4 h·ng/mL, 1210 ± 641 h·ng/mL, and 5030 ± 1960 h·ng/mL, respectively (Figure 10E). The C_max_ values for each group were 292 ± 78.2 ng/mL, 1450 ± 800 ng/mL, and 5800 ± 2150 ng/mL, respectively, while T_max_ reached 0.458 ± 0.102 h, 0.542 ± 0.246 h, and 0.458 ± 0.292 h, respectively (Figure 10E). The oral bioavailability was 26.7% at 1 mg/kg. No significant accumulation of ZSP1273 was observed in beagle dogs after 7 days of dosing at the 5 mg/kg dose twice daily *p.o.*, compared with day 1 systemic exposure (AUC_0-last_ and C_max_) (Figure 10F). ZSP1273 showed no sex differences at any of the dosage levels when comparing the AUC_0-last_ and C_0_/C_max_ in female and male beagle dogs.

After twice-daily oral administration of ZSP1273 at 3 mg/kg, 10 mg/kg, and 30 mg/kg in H1N1-infected female BALB/c mice for 7 consecutive days, the weight of mice in the 3 mg/kg group decreased continuously after viral infection and tended to become stable 3 days after administration, while the weights of mice in the 10 mg/kg and 30 mg/kg groups decreased continuously after viral inoculation and began to recover 3 days after administration (Figure 10G). The corresponding C_max_ values were 439 ng/mL, 3010 ng/mL, and 23,700 ng/mL, respectively, while the AUC_0-last_ values were 1040 ng·h/mL, 6190 ng·h/mL, and 56,000 ng·h/mL, respectively (Figure 10H). Following twice-daily oral administration of ZSP1273 at 3 mg/kg, 10 mg/kg, or 30 mg/kg in the H1N1-infected female BALB/c mice for 7 consecutive days, the corresponding C_max_ values were 439 ng/mL, 3010 ng/mL, and 23,700 ng/mL, respectively, while the AUC_0-last_ values were 1040 ng·h/mL, 6190 ng·h/mL, and 56,000 ng·h/mL, respectively. Within the dose range of 3 mg/kg to 30 mg/kg, both C_max_ and AUC_0-last_ increased with the increase in the dose (Figure 10H).

## 3. Discussion

Frequent mutations of HA and NA in the influenza A virus lead to decreases in its sensitivity to traditional antiviral drugs [22]. Moreover, the variable emergence of resistance to marketed drugs and the limited time window after infection within which these agents are active [19] mean that there is an urgent need to develop new anti-influenza drugs due to the limitations of approved agents. RNA polymerase is a pivotal enzyme regulating the replication of the influenza A virus [23,24]. The gene sequence that regulates the subunits of RNA polymerase is highly conserved, making it an attractive target for current anti-influenza-A drug research [25]. ZSP1273 is a novel RNA polymerase inhibitor that potentially targets the PB2 subunit. In this study, we found that ZSP1273 was effective against multiple strains of the influenza A virus, including oseltamivir-resistant strains, baloxavir-resistant strains, and highly pathogenic avian influenza viruses. Moreover, the antiviral efficacy of ZSP1273 on influenza A was evaluated in murine models and a ferret model.

RNA polymerase inhibitors are currently being studied for their ability to target the PA subunit and PB2 subunits. Favipiravir is the first broad-spectrum antiviral inhibitor targeting RNA polymerase to be approved for influenza treatment in Japan [26]. Baloxavir is an RNA polymerase inhibitor targeting the PA subunit, and it has also been approved as a clinical treatment for influenza A [21]. VX-787 is a novel influenza A virus PB2 inhibitor that has been proven to effectively inhibit influenza A virus infection in preclinical studies [27,28]. In this study, favipiravir, baloxavir, and VX-787 were used as reference compounds to evaluate the anti-influenza-A-virus activity of ZSP1273. In the structural design of ZSP1273, the introduction of cypropyl into position six of VX-787′s molecular pyrimidine ring can cleverly occupy the hydrophobic binding pocket, greatly improving the antiviral activity of the compound with the help of computer-aided drug design technology. In fact, the binding activity of ZSP1273 against influenza A polymerase has been confirmed in vitro. The research on ZSP1273′s inhibition of influenza A polymerase activity showed that the IC_50_ of ZSP1273 was 0.562 ± 0.116 nM, while the IC_50_ values of favipiravir and VX-787 were 34,087.67 ± 16,009.21 nM and 1.449 ± 0.34 nM, respectively, indicating that ZSP1273 had a better inhibitory effect on influenza A virus RNA polymerase activity than the inhibitor VX-787 (with the same target) and the pan-RNA polymerase inhibitor favipiravir. The non-target kinase assay, GPCR assay, and hERG assay confirmed that ZSP1273 has good biological safety. In addition, ZSP1273 showed no inhibitory activity against HBV, HCV, HRV, EV71, RSV, HPIV-3, or HSV-1, indicating the high specificity of ZSP1273 for influenza A virus infection.

The high mutagenicity of the influenza A virus has led to the emergence of multiple drug-resistant strains. During the 2008–2009 influenza season, more than 90% of seasonal influenza A (H1N1) viruses in many countries reported resistance to oseltamivir [29]. A previous study reported that oseltamivir resistance is mainly due to the H275Y mutation—a conformational change that occurs at the binding site of the neuraminidase inhibitor, preventing binding of oseltamivir [30]. The PI38T site mutation of the H1N1 and H3N2 virus strains caused by baloxavir reduced the sensitivity of the virus strain to baloxavir [21,31]. The PA I38T substitution is a major pathway for reduced susceptibility to Baloxavir, with 30-50-fold EC_50_ changes in the influenza A virus [32]. The emergence of viral strains that are resistant to these licensed drugs makes the development of new anti-influenza drugs particularly important. In this study, ZSP1273 exhibited high inhibitory activity against all tested influenza A virus strains, with mean EC_50_ values ranging from 0.012 to 0.063 nM. In comparison to the activity of oseltamivir against the wild-type influenza virus A/Weiss/43 (H1N1), which has been reported to be resistant to oseltamivir treatment, ZSP1273 demonstrated significant inhibitory activity. Furthermore, ZSP1273 exhibited an EC_50_ value of 0.028 nM against baloxavir-resistant influenza A/PR/8/34 (H1N1). The above results indicate that ZSP1273 could effectively inhibit influenza A virus infection, especially for oseltamivir-resistant and baloxavir-resistant virus strains. These results provide supporting data for the potential use of ZSP1273 in clinical treatment against influenza viruses—in particular, to address the problem of resistance to existing drugs. In vivo, murine models and ferret models of H1N1 virus infection were selected to analyze the preventive and therapeutic effects of ZSP1273 against influenza A. In the murine pharmacology studies, in terms of the time of administration, ZSP1273, oseltamivir, and VX-787 at doses of 10 mg/kg all improved the survival of mice within 4 h before infection or 48 h after infection. However, after 72 h of H1N1 infection, administration of ZSP1273 could only partially protect the virus-infected mice, while administration of oseltamivir (10 mg/kg) and VX-787 (10 mg/kg) exhibited no protective effect on the mice. Even 96 h after infection, ZSP1273 extended the time it took for all animals to die, indicating that ZSP1273 has a longer therapeutic window. Moreover, when conducting experiments on different species of ferrets, ZSP1273 performed more effectively than VX-787 in terms of virus growth inhibition, infection prevention, and nasal symptom remission in ferret models. These results further indicate that ZSP1273 has more advantages over VX-787 in terms of antiviral action.

Meanwhile, oseltamivir did not perform well in murine models, but ZSP1273 was better than VX-787 and oseltamivir in reducing viral titers within 3 days, and on the 5th day, ZSP1273 still had an inhibitory effect that was significantly greater than that of oseltamivir. Even if we used higher doses later, there were still four, five, and six deaths in the VX-787, oseltamivir, and favipiravir groups at 100 mg/kg, respectively, while only one animal died in the ZSP1273 group, suggesting that a high dose of ZSP1273 was still more effective and safer than oseltamivir. In the lethal model, the ZSP1273 high-dose and combination groups achieved zero mortality, which was better than the oseltamivir, VX-787, and baloxavir monotherapy groups. Meanwhile, in the non-lethal model, all administration groups except oseltamivir showed a significantly reduced lung index, and the viral titers in lung tissue were reduced to zero in all dosage groups of ZSP1273. Further, the combined dosage group, which achieved zero mortality, was significantly better than the oseltamivir monotherapy group. The results of these experiments show that ZSP1273 has very good efficacy and potential for combination use. The results of this series of studies once again demonstrate that ZSP1273 can effectively inhibit viral replication in vivo and successfully eliminate or attenuate influenza virus infection in mice.

Additionally, the problem of resistance to oseltamivir and baloxavir [10], along with adverse effects after the clinical use of oseltamivir—such as inhibiting the production of viral antigens, leading to a reduction in acquired antiviral humoral immunity and increasing the probability of reinfection [33]—mean that there is an urgent need for a new drug that is effective against drug-resistant strains and can be combined with oseltamivir to improve the efficiency of drug use.

Pharmacokinetic studies of ZSP1273 were conducted using SD rats, beagle dogs, and BALB/c mice. After single intravenous doses, ZSP1273 was slowly and moderately eliminated from SD rats and beagle dogs. After a single intragastric administration of ZSP1273 at different doses in SD rats and beagle dogs, the C_max_ and AUC_0-last_ of ZSP1273 were positively correlated with the administered doses, and there were no significant gender differences in the two parameters for rats and dogs. After repeated intragastric administration of ZSP1273, the C_max_ and AUC_0-last_ of male and female SD rats and beagle dogs showed no significant changes, and no accumulation was observed. In the in vivo study of BALB/c mice (infected with influenza A virus), after repeated oral gavage of ZSP1273, C_max_ and AUC_0-last_ were positively correlated with the dose level and were significantly higher than the increase in the dose ratio. These results indicate that ZSP1273 has good pharmacokinetic characteristics and is suitable for oral administration.

In conclusion, ZSP1273 is a novel influenza A virus RNA polymerase inhibitor. In vitro and in vivo models showed that ZSP1273 could effectively inhibit influenza A virus infection and exhibit high safety. ZSP1273 is a promising drug for the prevention and treatment of influenza A. The targeting of oseltamivir- and baloxavir-resistant viral strains will have more potential for development.

## 4. Materials and Methods

### 4.1. Chemicals, Viruses, and Cell Cultures

Chemicals: ZSP1273 was supplied by WuXi AppTec (Shanghai, China) Co., Ltd. and prepared as a 10 mM stock solution in 100% dimethyl sulfoxide (DMSO). VX-787 was supplied by WuXi AppTec (Shanghai, China) Co., Ltd. and prepared as a 10 mM stock solution in 100% dimethyl sulfoxide (DMSO). Favipiravir was supplied by Sichuan Nanbu Chengxin Technology Co., Ltd. (Nanchong, China). and prepared as a 10 mM stock solution in 100% dimethyl sulfoxide (DMSO). Oseltamivir was supplied by Toronto Research Chemicals and prepared as a 20 mM stock solution in 100% dimethyl sulfoxide (DMSO). Baloxavir was supplied by Shanghai Scochem Technology Co., Ltd. (Shanghai, China)—and prepared as a 20 mM stock solution in 100% dimethyl sulfoxide (DMSO).

Viruses: A/Anhui/01/2013 (H7N9) was obtained from the Chinese Center for Disease Control and Prevention; A/Qingyuan/GIRD01/2017 (H7N9) and A/Guangzhou/39715/2014 (H5N6) were clinical isolates, which were stored in the class III biosafety laboratory of the Guangdong Entry-Exit Inspection and Quarantine Bureau Inspection and Quarantine Technology Center. Influenza virus A/Mal/302/54 (H1N1), Influenza virus A/Hong Kong/8/68 (H3N2), influenza virus A/PR/8/34 (H1N1), influenza virus A/WS/33 (H1N1), influenza virus A/Weiss/43(H1N1), influenza virus B/Lee/40, influenza virus A/California/07/2009 (H1N1) pdm09, influenza virus A/California/2/2014 (H3N2), human rhinovirus (HRV), respiratory syncytial virus (RSV), herpes simplex virus 1 (HSV-1), and human parainfluenza virus type 3 (HPIV-3) were all purchased from the American Type Culture Collection (ATCC). Influenza virus A/WSN/33 (H1N1) was obtained from Virapur. Oseltamivir-resistant influenza virus strains A/Weiss/43 (H1N1)-4 and oseltamivir-resistant influenza virus strain A/Weiss/43 (H1N1)-5 were screened by WuXi AppTec (Shanghai) Co., Ltd. Human enterovirus 71 (EV71) was acquired from Xiamen University. Hepatitis C virus (HCV) replicon was constructed by WuXi AppTec (Shanghai) Co., Ltd. Hepatitis B virus (HBV) was obtained from the Wuhan Institute of Virology, Chinese Academy of Sciences. The baloxavir-resistant influenza A/PR/8/34 (H1N1) used in this experiment was provided by WuXi.

Cells: The Madin–Darby canine kidney (MDCK) cells were kindly donated by Prof. Malik Peiris of the University of Hong Kong. HEp-2 cells (ATCC) were cultured in DMEM/F12 medium containing 10% fetal bovine serum (FBS), 100 U/mL penicillin, and 100 g/mL streptomycin. H1 HeLa cells (ATCC) were cultured in DMEM medium supplemented with 3% FBS, 1% non-essential amino acid (NEAA), 100 U/mL penicillin, and 100 g/mL streptomycin. HepG2.2.15 cells (Wuhan Institute of Virology, Chinese Academy of Sciences) were maintained in DEME/F12 medium containing 2% FBS, 2 mM glutamine, 1% NEAA, 100 U/mL penicillin, and 100 g/mL streptomycin. Vero E6 cells (Wuhan Institute of Virology, Chinese Academy of Sciences) were cultured in DMEM medium supplemented with 2% FBS, 1% NEAA, 100 U/mL penicillin, and 100 g/mL streptomycin. Rhabdomyoma (RD) cells (Cell Bank of the Chinese Academy of Sciences, Shanghai, China) were cultured in DMEM medium containing 2% FBS, 1% sodium pyruvate, 1% NEAA, 2 mM glutamine, 100 U/mL penicillin, and 100 g/mL streptomycin. LLC-MK2 cells (Cell Bank of the Chinese Academy of Sciences, Shanghai, China) were maintained in DMEM medium containing 2% FBS, 1% NEAA, 2 mM glutamine, 100 U/mL penicillin, and 100 g/mL streptomycin. HCV genotype 1b (GT-1b) replicon cells were constructed by WuXi AppTec (Shanghai) Co., Ltd., and cultured in DMEM medium containing 10% FBS, 1% NEAA, 2 mM glutamine, 100 U/mL penicillin, 100 g/mL streptomycin, and 250 g/mL G418. The 293T cells, provided by Professor Chen Honglin from the University of Hong Kong, were maintained in DMEM medium with 10% FBS and 300 μg/mL G418. CHO cells were cultured in DMEM/F12 medium with 10% FBS, 300 μg/mL G418, and 2 μg/mL blasticidin. MDCK cells (ATCC) were cultured in EMEM medium supplemented with 10% FBS, 2 mM L-glutamine, 1% NEAA, 100 U/mL penicillin, and 100 g/mL streptomycin. OptiPRO SFM supplemented with 2 mM L-glutamine, 1% NEAA, 100 U/mL penicillin, 100 g/mL streptomycin, and 2.5 μg/mL trypsin was used as the assay medium.

### 4.2. Inhibition Assay of Influenza Virus Polymerase

The *PB1*, *PB2*, *NP*, and *PA* genes from A/California/04/2009 (H1N1) pdm09 were subcloned into a phw2000 plasmid. A pPHY luciferase plasmid (Promega) and a Renilla plasmid were used as a polymerase activity reporter and a transfection efficiency control, respectively. The HEK293T cells were seeded in a 12-well plate at a density of 250,000 cells per well for later use. An aliquot of 3 μL of EndoFectin Lenti Transfection Reagent was pipetted into an EP tube containing 50 μL of Opti-MEM, mixed well, and kept at room temperature for 10 min. Appropriate amounts of plasmids (PA-phw2000, PB1-phw2000, PB2-phw2000, NP-phw2000, pPHY luciferase plasmid, and Renilla plasmid) were transferred into an EP tube containing 50 μL of Opti-MEM, mixed well, and kept at room temperature for 10 min. The transfection reagent mixture in the EP tube was transferred into the EP tube containing the mixture of plasmids, mixed well, and kept at room temperature for 20 min. The mixed solution was transferred into the 293T cell culture plate and cultured in an incubator at 37 °C with 5% CO_2_. After a 6-h transfection, ZSP1273 (0.457–1000 nM), VX-787 (0.0001–1000 nM), and favipiravir (0.0456–100 μM) were added to the cell wells. After 24 h, the polymerase activity in the cells was measured by luciferase assay. Luciferase assay: First, we removed the supernatant of the cell liquid, washed the cell plate once with 1× PBS, added 1× LYSIS to lyse the cells, and transferred 20 μL of cell lysate to the 96-well plate. Finally, we added 100 μL of luciferase assay reagent to the mixture, mixed it well, and read the firefly luciferase (Fluc) values on a full-wavelength microplate reader. Then, we added 100 μL of Stop & Glo^®^ reagent (Promega, Tokyo, Janpan, Cat#E1910)to the mixture, mixed it well, and read the Renilla luciferase (Rluc) values on a full-wavelength microplate reader. The IC_50_ values were calculated via the Reed–Muench method.

### 4.3. Human Kinase Activity Inhibition Assay

Kinase activity was measured by Z’-LYTE^®^ technology (Invitrogen) based on the principle of fluorescence resonance energy transfer (FRET). ZSP1273, VX-787, and staurosporine solution were diluted by an automated liquid handling platform (Bravo, Agilent G5409A). Aliquots of 2.5 μL of the diluted compound solutions were transferred to the assay plate and then centrifuged at 1000 rpm for 1 min. The compounds were diluted from 10,000 nM to 0.17 nM in the final reaction system. An aliquot containing a 5 μL kinase and peptide mixture in assay buffer (50 mM HEPES pH 7.5, 1 mM EGTA, 10 mM MgCl_2_, 0.01% Brij-35) was added to the assay plate, and then the plate was incubated at 23 °C for 15 min. Subsequently, an aliquot of 2.5 μL of ATP solution was added to the assay plate and centrifuged at 1000 rpm for 1 min. CK1alpha and MAP4K4 were incubated for 90 min, TNIK for 120 min, and other kinases for 60 min. An aliquot of 5 μL of assay reagent was added to the assay plate and centrifuged at 1000 rpm for 1 min, and then the plate was incubated at 23 °C for 60 min before being read on an Envision multilabel plate reader. The IC_50_ values were calculated using XLfit5 (formula 205) developed by IDBS. The assay reaction conditions of ZSP1273 against 24 kinases are shown in Table 4.

### 4.4. Evaluation of G-Protein-Coupled Receptors’ Activity

The reference compounds are shown in Table A3 and Table A4. Twenty-two cell lines stably expressing the relevant receptors were constructed by WuXi AppTec (Shanghai, China) Co., Ltd. Specific information and culture conditions are listed in Table A3 and Table A4. The cells were seeded into 384-well polylysine-coated cell plates at a density of 20,000 cells per well (20 μL), and then they were incubated overnight at 37 °C in a 5% CO_2_ incubator. The test and reference compounds were diluted with 100% DMSO, and gradient dilution compound plates were prepared with ECHO. The cell plates were then supplemented with 20 μL of 2× Fluo-4 Direct^TM^ buffer per well and incubated in a 5% CO_2_ incubator at 37 °C for 50 min. After the cells were kept at room temperature for 10 min, 10 μL of experimental buffer salt solution was added to the cell plates to read the fluorescence signal. Then, 10 μL of a reference compound agonist was added to the plates to read the fluorescence signal, and the EC_80_ of the corresponding GPCR target was calculated. To read the fluorescence signal according to the set program, 10 μL of the test compound and the reference compounds were added to the cell plates at detectable concentrations. In order to read the fluorescence signal, 10 μL of the reference compound agonist with a concentration of 6× EC_80_ was added to the cell plates. The compound agonist assay data were exported by means of “Max–Min” and “Read 1 to 90” using the software. The compound antagonist assay data were exported by means of “Max–Min” and “Read 90 to Maximum allowed” using the software. The data were analyzed using Prism statistical software.

### 4.5. Cytopathic Effect (CPE) Inhibition Assay

In this study, the CPE method was used to evaluate the in vitro inhibitory effect of ZSP1273 on the replication of various influenza A virus strains (H1N1 and H3N2), oseltamivir-resistant strains, baloxavir-resistant virus strains, positive-strand RNA viruses (human rhinovirus (HRV) and human enterovirus 71 (EV71), negative-strand RNA viruses (respiratory syncytial virus (RSV) and human parainfluenza virus type 3 (HPIV-3), and a DNA virus (herpes simplex virus type 1 (HSV-1)), along with combination experiments of ZSP1273 with oseltamivir. MDCK cells were seeded in 96-well cell culture plates at a density of 15,000 cells per well and cultured at 37 °C with 5% CO_2_ overnight. The next day, serially diluted compounds (3-fold serial dilution, eight concentrations, in triplicate wells) and viruses were added, with ZSP1273, VX-787, and oseltamivir at concentration ranges of 1–0.0005 nM, 10–0.0046 nM, and 100–0.0457 µM, respectively. The final concentration of DMSO in the cell culture medium was 0.5%. The resulting cultures were incubated for an additional 2–5 days. Cell viability was detected by CellTiter-Glo or CCK-8 according to the manufacturer’s instructions. The cytotoxicity of the compounds was assessed in parallel under the same conditions, but without viral infection. MDCK cells were seeded in 96-well cell culture plates at a density of 15,000 cells per well and cultured at 37 °C and 5% CO_2_ overnight. Next day, serially diluted compounds (3-fold serial dilution, 8 concentrations, in triplicate wells) with the concentration ranges of ZSP1273, VX-787, and Oseltamivir of 100–0.0457 µM. The final concentration of DMSO in the cell culture medium was 0.5%. The resulting cultures were incubated for additional 2–5 days. Cell viability was detected by CellTiter-Glo or CCK-8 following the manufacturer’s manual. The antiviral activity and cytotoxicity of the compounds were expressed as % inhibition and % viability based on the protection against the virus-induced CPE, respectively. The raw CPE data indicate the values of the compound-treated wells; average VC, average CC, and average MC indicate the average values of the virus control, cell control (i.e., cells without viral infection or compound treatment), and medium control (i.e., medium only) wells, respectively. The EC_50_ and CC_50_ values were calculated using GraphPad Prism 6.0.

### 4.6. Evaluation of ZSP1273 against Highly Pathogenic Avian Influenza Viruses H7N9 and H5N6

MDCK cells were seeded in a 96-well plate for culture. The serum-free, DMEM-diluted viruses (10-1-10-10) were added to the 96-well plate (100 μL/well, repeated for 6 wells), and a blank control group was set up at the same time. After discarding the supernatant, the plate was incubated at 37 °C in a 5% CO_2_ incubator for 2 h and washed twice with 1× PBS. The basal medium DMEM was then added to continue the incubation for another 48 h. The cell growth was observed daily, and when the cells showed cellular atrophy, rounding, and exfoliation, the occurrence of CPE was recorded, and the TCID_50_ was calculated via the Reed–Muench method. The ZSP1273 was serially diluted in 5 gradients, with the high-dose concentration (0.5 ng/mL) as the initial concentration. Each group was inoculated with a highly pathogenic avian influenza virus solution (with a viral titer of 100 TCID_50_) at 100 μL/well. An equal amount of diluted virus solution was added to the normal group, and the supernatant was discarded after adsorption for 2 h. Corresponding dilutions of drug were added to the drug group, while the normal group and the virus group were supplemented with an equal amount of drug diluent for incubation for 48 h. Then, 20 μL of MTT solution (5 mg/mL, i.e., 0.5% MTT) was added, and the incubation was continued for another 4 h. Next, 150 μL of DMSO was added to each well. The absorbance of each well was measured at OD 490 nm on an ELISA instrument. The 50% inhibitory concentration (IC_50_) was calculated via the Reed–Muench method.

The in vitro cytotoxicity of the compounds was assessed in parallel under the same conditions, but without viral infection. After the MDCK cells formed a monolayer in the 96-well plate for culture and the plate was washed with PBS, WXFL20100273 at an initial concentration of 25 μg/mL was diluted 2-fold to set seven gradients, and then it was added to the wells at 100 μL/well (four duplicate wells); a blank group (with medium only, without cells) and a normal cell control group (with drug dissolution medium) were also set up simultaneously. The cells were incubated with 5% CO_2_ at 37 °C for 48 h, and then 20 μL of MTT solution was added (5 mg/mL, i.e., 0.5% MTT). The incubation lasted for another 4 h until termination. Then, 150 μL of DMSO was added to each well, and the plate was placed on a shaker for 10 min at low speed. The absorbance at 490 nm in each well was measured on a full-wavelength scanner, and the TC_50_ and TC_0_ of the drug were calculated. The selective index (SI) was calculated using the formula SI = TC_50_/IC_50_. The assay was repeated three times.

### 4.7. HCV Replicon Assay

The compounds were serially diluted using the ECHO555 Liquid Workstation System and added to 96-well plates. HCV GT-1b replicon cells were then seeded into a 96-well experimental plate containing the compounds at a density of 8000 cells per well with a final DMSO concentration of 0.5%. The cells were incubated in an incubator at 5% CO_2_ and 37 °C for 3 days. The cell viability was measured using the CellTiter-Fluor Cell Vitality Assay Kit to determine the cytotoxicity of the compounds. The luciferase activity in the HCV replicons was detected using Britelite plus—a luciferase luminescence substrate—and the data were used to determine the inhibitory activity of the compound against HCV replicons.

A nonlinear fitting analysis of compound inhibition and viability was performed using the GraphPad Prism (version 5) software to determine the EC_50_ and CC_50_ of the compounds.

### 4.8. Anti-Hepatitis-B-Virus Assay

The anti-HBV activity was detected in HepG2.2.15 cells, which are stable HBV-transfected cells that consistently express HBV and are commonly used to determine the anti-HBV activity of compounds. HepG2.2.15 cells were seeded into 96-well cell culture plates at a density of 40,000 cells per well, followed by the addition of diluted compounds (3× serial dilution, 8 concentration points, in triplicate). Entecavir (ETV) was used as a positive control. The final concentration of DMSO in the cell medium was 0.5%. The HepG2.2.15 cells were incubated at 37 °C with 5% CO_2_. The medium was replaced with fresh medium containing the same concentration of compound on the third day, and the supernatant was collected on the sixth day. The HBV DNA was extracted from the supernatant using the QIAamp 96 DNA Blood Kit and quantified by quantitative real-time polymerase chain reaction (qPCR). The quantified standard HBV plasmid DNA was diluted in a 3-fold series to 8 concentration points and subsequently added to the qPCR reaction system with the sample DNA (10 μL each) for PCR. A standard curve was plotted using the CT value of standard plasmid DNA to quantify the numbers of HBV DNA copies in the sample wells and control wells. The cytotoxicity assay was performed in parallel with the antiviral assay under the same conditions. The cell viability was detected using the CellTiter-Glo assay. A nonlinear fitting analysis of compound inhibition and viability was performed using GraphPad Prism (version 5) software to determine the EC_50_ and CC_50_ of the compounds.

### 4.9. hERG Potassium Channel

ZSP1273-mediated inhibition of the hERG potassium channel (human Ether-a-go-go-related gene potassium channel) was measured by the electrophysiological manual patch-clamp method. When testing the CHO (Chinese Hamster Ovary) cells with stable expression of the hERG potassium channel, the hERG potassium channel current was recorded at room temperature using the whole-cell patch-clamp technique. The clamping voltage and data were controlled and recorded using the pCLAMP 10 software with a sampling frequency of 10 kHz and a filtering frequency of 2 kHz. After the whole-cell recordings were obtained, the clamp voltage was at −80 mV, which triggered the step voltage of the hERG potassium current (hERG) from −80 mV (applied with a depolarization voltage for 2 s) to +20 mV, followed by depolarization to −50 mV for 1 s, and then down to −80 mV. This voltage stimulation was administered every 10 s, and the drug administration was initiated after the hERG potassium current was stabilized (for 1 min). The compound was continuously administered at the lowest concentration, and each concentration lasted for 1 min. At least 3 cells (n ≥ 3) were tested at each concentration. Briefly, ZSP1273 was serially diluted 3-fold for 6 concentrations (the concentration ranges were 0.16–40 µM). Cisapride was serially diluted 3-fold for 5 concentrations (the concentration ranges were 3.70–300 nM). The GraphPad Prism (version 8) software was used to determine the IC_50_ values of the compounds.

### 4.10. Animals

The female BALB/c mice (specific-pathogen-free (SPF) grade, 6–7 weeks old, 16–18 g) used in the murine infection experiments and pharmacokinetic studies were purchased from Shanghai Lingchang Biotech Co., Ltd. (Shanghai, China). The ferrets (*Mustela Putorius Furo*, female, 10–16 weeks old, fixed and de-scented), purchased from Wuxi Sangosho Biotechnology Co., Ltd., (Wuxi, China) had been confirmed as negative for influenza virus A/California/04/2009 serum antibodies by a micro-neutralization assay. The SD rats were supplied by Beijing Vital River Laboratory Animal Technology Co., Ltd., Beijing, China Male SD rats with certificate number 11400700256361 were received on 20 October 2017; female SD rats with certificate number 11400700255558 were received on 17 October 2017. The beagle dogs used in this study were supplied by Marshall Bioresources (Beijing, China). The animals were confirmed to be healthy by WuXi veterinarians before being assigned to the study. Each animal was given a unique identification number marked on the ear and written on the cage card. According to the IACUC, a mouse dying or losing more than 35% of its weight (based on the weight on the day of infection) would be considered dead (humanitarian endpoint). The laboratory animal quality certificate number was S(X)FXF 2014004. All animal care and experimental procedures were approved by the Institutional Animal Care and Treatment Committee of WuXi AppTec (approval numbers: R20160317-Mouse and N20170303).

### 4.11. Efficacy Experiments with ZSP1273 in the Murine Infection Model

#### 4.11.1. Murine Infection Experiment I

The 60 mice were divided into 10 groups, with six mice in each group. Groups 1 and 2 were gavaged with the vehicle; Groups 3 and 4 were gavaged with oseltamivir (10 mg/kg); Groups 5–8 were gavaged with ZSP1273 (3 mg/kg, 10 mg/kg, 10 mg/kg, and 30 mg/kg); and Groups 9 and 10 were gavaged with VX-787 (10 mg/kg). The mice in Groups 1, 3, 6, and 9 (euthanatized on day 3) were gavaged twice daily on days 1–2, while those in Groups 2, 4, 5, 7, 8, and 10 (euthanatized on day 5) were gavaged twice daily on days 1–4; the administration interval was 10 h/14 h. The first dose was administered at 36 h after infection with influenza A virus WSN/33 (H1N1) (inoculation size of 1.0 × 10^4^ PFU/mouse). On day 3 or day 5—the two endpoints of the in vivo study—all of the mice were euthanatized, while their lung tissues were collected and quickly frozen in DPBS at 10 times the lung volume for the viral plaque assay [27]. From day 0 to day 5, the weight of the mice was monitored daily.

#### 4.11.2. Murine Infection Experiment II

The 104 mice were divided into 13 groups, with 8 mice in each group. Group 1 was gavaged with the vehicle, Groups 2–5 were gavaged with oseltamivir (10 mg/kg), Groups 6–9 were gavaged with ZSP1273 (10 mg/kg), and Groups 10–13 were gavaged with VX-787 (10 mg/kg). The mice in Groups 1, 2, 6, and 10 were gavaged twice daily from day 0 to day 6, with the first dose given at 4 h pre-infection; the mice in Groups 3, 7, and 11 were gavaged twice daily from day 2 to day 8, with the first dose given at 48 h after infection; the mice in Groups 4, 8, and 12 were gavaged twice daily from day 3 to day 9, with the first dose at 72 h after influenza A virus WSN/33 (H1N1) infection (inoculation size of 1.0 × 10^4^ PFU/mouse), while those in Groups 5, 9 and 13 were gavaged twice daily from day 4 to day 10, with the first dose at 96 h after infection. The administration interval was 10 h/14 h. From day 0 to day 14, the weight and viability of the mice were monitored daily. Day 14 was the endpoint of the study, at which point all of the living mice were euthanatized after being weighed.

#### 4.11.3. Murine Infection Experiment III

The 64 mice were divided into 8 groups, with 8 mice in each group. Group 1 was gavaged with the vehicle; Groups 2 and 3 were gavaged with oseltamivir (30 mg/kg and 100 mg/kg); Groups 4 and 5 were gavaged with ZSP1273 (30 mg/kg and 100 mg/kg); Groups 6 and 7 were gavaged with VX-787 (30 mg/kg and 100 mg/kg); and Group 8 was gavaged with Favipiravir (100 mg/kg). From day 2 to day 8, all of the animals were orally gavaged twice daily. The first dose was administered 48 h after influenza A virus WSN/33 (H1N1) infection; the administration interval was 10 h/14 h. From day 0 to day 14, the weight and viability of the mice were monitored daily. Day 14 was the endpoint of the study, at which point all of the living mice were euthanatized after being weighed.

#### 4.11.4. Murine Infection Experiment IV

BALB/c mice were intranasally infected with the 2LD_50_ (lethal dose) influenza virus. The 90 mice were divided into 9 groups, with 10 mice in each group. The drug groups (ZSP1273 30 mg/kg, ZSP1273 10 mg/kg, ZSP1273 3 mg/kg, VX-787 30 mg/kg, baloxavir 30 mg/kg, oseltamivir 30 mg/kg, and ZSP1273 3 mg/kg + oseltamivir 30 mg/kg combined administration group), blank control group, and virus control group were set. From day 3 to day 7, all of the animals were orally gavaged twice daily. The first dose was administered 72 h after A/PR/8/34 (H1N1) infection (inoculation size of 2.0 × 10^5^ PFU/mouse); the administration interval was 10 h/14 h. From day 0 to day 16, the weight and viability of the mice were monitored daily. Day 14 was the endpoint of the study, at which point all of the living mice were euthanatized after being weighed.

#### 4.11.5. Murine Infection Experiment V

BALB/c mice were intranasally infected with the LD_50_ (non-lethal dose) influenza virus. The 90 mice were divided into 9 groups, with 10 mice in each group. The drug groups (ZSP1273 30 mg/kg, ZSP1273 10 mg/kg, ZSP1273 3 mg/kg, VX-787 30 mg/kg, baloxavir 30 mg/kg, oseltamivir 30 mg/kg, and ZSP1273 3 mg/kg + oseltamivir 30 mg/kg combined administration group), blank control group, and virus control group were set. From day 3 to day 7, all of the animals were orally gavaged twice daily. The first dose was administered 72 h after A/PR/8/34 (H1N1) infection (inoculation size of 1.0 × 10^5^ PFU/mouse); the administration interval was 10 h/14 h. From day 0 to day 8, the weight and viability of the mice were monitored daily. Day 8 was the endpoint of the study, at which point all of the living mice were euthanatized after being weighed, and the lung tissue of the mice was aseptically harvested and weighed. The lung index of the mice was calculated, and the viral titers of the lung tissue were measured.

### 4.12. Antiviral Activity of ZSP1273 in the Ferret Infection Model

The 19 ferrets were divided into 4 groups: the vehicle group (n = 5), the oseltamivir group (25 mg/kg/day, n = 4), the VX-787 group (25 mg/kg/day, n = 5), and the ZSP1273 group (25 mg/kg/day, n = 5). The vehicle group was gavaged twice daily with 5 mL/kg of 10% Kolliphor^®^ HS15 solution, and the other groups were gavaged twice daily with 25 mg/kg/day of the corresponding drugs. The interval between the two doses was never less than 6 h or more than 12 h, with the first dose administered 4 h before influenza A virus/California/07/2009 (H1N1) infection (inoculation size of 1.0 × 10^5^ PFU/ferret). The administration was performed for five consecutive days. The weights of the animals were measured and recorded before infection. After the animals were anesthetized by intraperitoneal injection of pentobarbital sodium (40 mg/kg), a 1.0 × 10^5^ TCID_50_ (200 μL) virus solution was dropped into their nostrils. Nasal lavage: After the body weights were recorded on days 2, 4, and 6, the animals were anesthetized by intraperitoneal injection of pentobarbital sodium (40 mg/kg). In the safety cabinet, the animal lay in a lateral position, and 1 mL of sterile PBS (containing 400 U of penicillin, 400 μg/mL of streptomycin, and 5 μg/mL of amphotericin B) was dropped into one nostril. The lavage liquids were dropped onto a 100-mm plate and collected into a centrifuge tube, followed by storage on ice. Then, the viral titer of the lavage fluid was determined. The weight and viability of the ferrets were monitored daily. These observations were continued for 3 days until no symptoms were observed in the vehicle group. The weight of the ferrets in the vehicle group returned to pre-infection levels and remained stable for more than 3 days after day 12, so day 15 was the endpoint of the study. During the experiment, 1 ferret (No. 10) in the oseltamivir group died on day 2, without any abnormalities being found in the autopsy. The death of this animal was defined as a nonspecific death from unknown causes, with all associated data excluded from the statistics.

### 4.13. Pharmacokinetic Study of Single- and Multi-Dose ZSP1273 Given by Oral or Intravenous Administration in Rats, Beagle Dogs, and Mice

A sensitive, specific, and reproducible LC–MS/MS method for the quantitative determination of ZSP1273 in the plasma of SD rats, beagle dogs, and female BALB/c mice was developed and validated. The dynamic range of the method was 2.00–5000 ng/mL (female BALB/c mice) and 5.00–10,000 ng/mL (SD rats and beagle dogs).

Thirty (30; 15/sex) SD rats were divided into five groups, with three animals/sex/group. Animals in Group 1 were given ZSP1273 by a single intravenous bolus administration at 3 mg/kg. The vehicle used for the IV study was DMSO:Solutol:water (5:5:90, *v*/*v*/*v*). Animals in Groups 2–4 were given ZSP1273 by a single oral administration at 3, 15, and 75 mg/kg, respectively. Animals in Group 5 were given ZSP1273 by oral administration twice daily at 15 mg/kg for 7 consecutive days. The vehicle used for the oral studies was 20% (*v*/*v*) Solutol in water, with a dose volume of 10 mL/kg. Plasma samples were collected at pre-dose (0), and at 0.083, 0.25, 0.5, 1, 2, 4, 6, 8, 12, and 24 h post-dose for Groups 1–4. For Group 5, plasma was collected at pre-dose (0) and at 0.083, 0.25, 0.5, 1, 2, 4, 8 (before the second dose), 8.25, 9, 12, 16, and 24 h post-dose on days 1 and 7. The concentrations of ZSP1273 in the plasma samples were determined by a validated liquid chromatography–tandem mass spectrometry (LC–MS/MS) method.

Thirty (30; 15/sex) naïve beagle dogs were divided into five groups, with three animals/sex/group. Animals in Group 1 were given ZSP1273 by a single intravenous bolus administration at 1 mg/kg. Animals in Groups 2–4 were given ZSP1273 by a single oral administration at 1, 5, and 15 mg/kg, respectively. Animals in Group 5 were given ZSP1273 by oral administration twice daily (the dosing interval was 8 h) at 5 mg/kg for 7 consecutive days. Plasma samples were collected at pre-dose (0) and at 0.083, 0.25, 0.5, 1, 2, 4, 6, 8, 12, and 24 h post-dose for Groups 1–4. For Group 5, plasma was collected at pre-dose (0) and at 0.083, 0.25, 0.5, 1, 2, 4, 8 (before the second dose), 8.25, 9, 12, 16, and 24 h post-dose on days 1 and 7. The concentrations of ZSP1273 in the plasma were determined by the liquid chromatography–tandem mass spectrometry (LC–MS/MS) method.

Twenty-seven (27) female BALB/c mice were divided into three groups, with nine animals per group. Animals in Groups 1–3 were given ZSP1273 by oral administration at 3, 10, and 30 mg/kg, respectively, for 7 consecutive days, twice daily. Plasma samples were collected at pre-dose (0) and at 0.25, 1, 4, 7, 10 (before the second dose), 10.25, 11, and 14 h post-dose on day 7. The weight of the mice was monitored daily. The concentrations of ZSP1273 in the plasma were determined by the liquid chromatography–tandem mass spectrometry (LC–MS/MS) method.

## Figures and Tables

**Table 1:** Inhibitory activity of ZSP1273 against multiple influenza virus strains.

Virus Strain/Cell	ZSP1273	VX-787	Oseltamivir
	(nM)	(nM)	(μM)
Influenza virus A/Mal/302/54 (H1N1) *	0.028 ± 0.011	0.295 ± 0.111	1.341 ± 1.915
Influenza virus A/Hong Kong/8/68 (H3N2) *	0.019 ± 0.010	0.162 ± 0.034	0.019 ± 0.010
Influenza virus A/PR/8/34 (H1N1) *	0.042 ± 0.010	0.760 ± 0.355	4.889 ± 7.371
Influenza virus A/WS/33 (H1N1) *	0.032 ± 0.010	0.206 ± 0.061	2.429 ± 1.240
Influenza virus A/WSN/33 (H1N1) *	0.063 ± 0.028	0.315 ± 0.070	10.067 ± 9.820
Influenza virus A/Weiss/43(H1N1) *	0.012 ± 0.002	0.255 ± 0.055	5.618 ± 2.779
Influenza virus B/Lee/40 *	>1 ***	>10 ****	2.937 ± 3.619
MDCK (33 °C) **	1.632 ± 0.870	21.066 ± 9.764	>100 *****
MDCK (37 °C) **	1.777 ± 0.774	28.888 ± 6.125	>100 *****

Note: * The data are presented as EC_50_ (mean ± SD) measured by the cytopathic effect method in MDCK cells, and the inhibitory activity of the compounds was analyzed by nonlinear fitting using GraphPad Prism (Version 5) software to calculate the EC_50_. ** Data are presented as CC_50_ (mean ± SD) measured by cytotoxicity experiments in MDCK cells, and the cell viability of the compounds was analyzed by nonlinear fitting using GraphPad Prism (Version 5) software to calculate the CC_50_. *** ZSP1273 was tested at a maximum concentration of 1 nM. **** VX-787 was tested at a maximum concentration of 10 nM. ***** Oseltamivir was tested at a maximum concentration of 100 μM.

**Table 2:** Inhibitory activity of ZSP1273 against drug-resistant strains and highly pathogenic avian influenza viruses.

Virus Strain/Cell	ZSP1273	VX-787	Oseltamivir	Baloxavir
	(nM)	(nM)	(nM)	(nM)
Oseltamivir-resistant influenza virus strain A/Weiss/43 (H1N1)-4 ^a^	0.014 ± 0.006	0.234 ± 0.094	>100,000 *	ND **
Oseltamivir-resistant influenza virus strain A/Weiss/43 (H1N1)-5 ^a^	0.017 ± 0.004	0.216 ± 0.053	>100,000	ND
Baloxavir-resistant influenza A/PR/8/34 (H1N1) ***^a^	0.028 ± 0.013	0.434 ± 0.052	ND	89.440 ± 11.866
A/Anhui/01/ 2013(H7N9) ^b^	0.627 ± 0.312	0.834 ± 0.204	175,394.95 ± 3791.52	ND
A/Qingyuan/ GIRD01/2017 (H7N9) ^b^	0.777 ± 0.427	1.916 ± 0.492	1,688,467 ± 760,314	ND
A/Guangzho u/39715/2014 (H5N6) ^b^	0.245 ± 0.03	0.0918 ± 0.0145	1,654,747 ± 1,222,172	ND

Note: * Oseltamivir was tested at a maximum concentration of 100,000 nM. ** ND: not tested. *** The mutation site of the baloxavir-resistant influenza virus strain A/PR/8/34 (H1N1) is PA I38T. ^a^ Data are presented as EC_50_ (mean ± SD) measured by the cytopathic effect method in MDCK cells, and the inhibitory activity of the compounds was analyzed by nonlinear fitting using GraphPad Prism (Version 5) software to calculate the EC_50_. ^b^ Data are presented as IC_50_ (mean ± SD) measured by the cytopathic effect method in MDCK cells, which was calculated by the Reed–Muench method.

**Table 3:** Assay results of ZSP1273 against different viruses.

Virus/Cell	Compound	EC_50_ for Virus *	CC_50_ for Cell *
RSV long/Hep-2 ^a^	ZSP1273	>10 μM	>10 μM
	BMS-433771	0.014 μM	>1 μM
HSV-1 GHSV-UL46/Vero E6 ^a^	ZSP1273	>10 μM	>10 μM
	Acyclovir	0.655 μM	>10 μM
EV71 Shenzhen/120F1/09/RD ^a^	ZSP1273	>10 μM	>10 μM
	AG7088	0.012 μM	>1 μM
HPIV-3 C243/LLC-MK2 ^a^	ZSP1273	>10 μM	>10 μM
	Ribavirin	20.310 μM	>100 μM
HRV 1B/H1 HeLa ^a^	ZSP1273	7.88 μM	>10 μM
	Pleconaril	0.028 μM	>5 μM
HBV HepG2.2.15 ^b^	ZSP1273	>10 μM	>10 μM
	Entecavir	0.338 nM	>20 nM
HCV replicon 1b ^c^	ZSP1273	>10 μM	>10 μM
	BMS-790052	0.021 nM	>3 nM

Note: * ZSP1273 was tested at a maximum concentration of 10 μM. The maximum tested concentrations of BMS-433771, acyclovir, AG7088, ribavirin, pleconaril, entecavir, and BMS-790052 were 1 μM, 10 μM, 1 μM, 100 μM, 5 μM, 20 nM, and 3 nM, respectively. A nonlinear fitting analysis of compound inhibition and viability was performed using GraphPad Prism (version 5) software to determine the EC _50_ and CC_50_ of the compounds. ^a^ The five virus assays were carried out using the cytopathic effect (CPE) method induced by viral infection to determine the antiviral activity of WXFL20100273. ^b^ The hepatitis B virus assay was performed using the quantitative PCR (qPCR) method on the HepG2.2.15 cell line, with stable transfection of HBV expression. ^c^ The hepatitis C virus assay was performed using hepatitis C virus genotype 1b replicon cells.

**Table 4:** Assay reaction conditions of ZSP1273 against 24 kinases.

Kinase	Enzyme (nM)	Substrate (µM)	ATP (µM)	Substrate
ABL1	0.8	2	14	Tyr 2 substrate
Aurora A	1.5	2	12	Ser/Thr 1 substrate
CAMK2 alpha	2	2	40	Ser/Thr 4 substrate
CDK2	3	2	34	Ser/Thr 12 substrate
CHK1	8	2	58	Ser/Thr 19 substrate
CK1 alpha	15	2	2	Ser/Thr 11 substrate
DYRK3	3.2	2	5	Ser/Thr 9 substrate
EPHA2	10	2	25	Tyr 1 substrate
FGFR1	1.5	2	10	Tyr 4 substrate
GSK3 beta	1.5	2	6	Ser/Thr 9 substrate
JAK2	1.2	2	27	Tyr 6 substrate
JNK1	3	2	100	Ser/Thr 4 substrate
KDR	3	2	100	Tyr 1 substrate
LCK	4	2	80	Tyr 2 substrate
MAP4K4	1.25	2	13	Ser/Thr 7 substrate
MAPKAPK2	0.8	2	3	Ser/Thr 4 substrate
MINK	1.25	2	50	Ser/Thr 7 substrate
MST4	10	2	28	Ser/Thr 7 substrate
p38 alpha	7.5	2	298	Ser/Thr 15 substrate
PDK1	5	2	100	Ser/Thr 6 substrate
PKA	0.5	2	6	Ser/Thr 1 substrate
SRC	6	2	60	Tyr 2 substrate
TAOK2	4	2	153	Ser/Thr 7 substrate
TNIK	35	2	64	Ser/Thr 7 substrate

## Data Availability

The data presented in this study are available upon request from the corresponding author.

## Abbreviations

AUC: area under the plasma concentration–time curve; ATCC: American Type Culture Collection; b.i.d.: bis in diē; CHO: Chinese hamster ovary; CPE: cytopathic effect; C_max_: maximum plasma concentration; CCK-8: Cell Counting Kit-8; CC_50_: 50% cytotoxic concentration; CT: cycle threshold; CL: systemic clearance; DMSO: dimethyl sulfoxide; DMEM: Dulbecco’s Minimal Essential Medium; DPBS: Dulbecco’s Phosphate-Buffered Saline; d.p.i.: days post-infection; EC_50_: concentration for 50% of maximal effect; EC_80_: concentration for 80% of maximal effect; EC_90_: concentration for 90% of maximal effect; ETV: entecavir; EV71: human enterovirus 71; FBS: fetal bovine serum; FLUC: firefly luciferase; FRET: fluorescence resonance energy transfer; GPCRs: G-protein-coupled receptors; HA: hemagglutinin; hERG: human ether-a-go-go-related gene; HRV: human rhinovirus; HCV: hepatitis C virus; HPIV-3: human parainfluenza virus type 3; HSV-1: herpes simplex virus 1; HBV: hepatitis B virus; IC_50_: 50% inhibitory concentration; IACUC: humanitarian end point; *i.v.*: intravenous injection; LC–MS: liquid chromatography–mass spectrometry; LLOQ: lower limit of quantification; MDCK: Madin–Darby canine kidney; MTT: 3-(4,5-dimethylthiazol-2-yl)-2,5-diphenyltetrazolium bromide; MEM: Minimum Essential Medium; MRM, multiple reaction monitoring; MsOH: methanesulfonic acid; NA: neuraminidase; NEAA: non-essential amino acid; NMR: nuclear magnetic resonance; Opti-MEM: Opti-MEM^®^ I Reduced Serum Medium; OD: optical density; PB1: polymerase basic protein 1; PB2: polymerase basic protein 2; PA: polymerase acidic protein; PBS: phosphate-buffered saline; PD: pharmacodynamics; PDB: Protein Data Bank; PK: pharmacokinetics; *p.o.*: per os; PFU: plaque-forming unit; qPCR: quantitative real-time polymerase chain reaction; QC: quality control; RSV: respiratory syncytial virus; RD: rhabdomyoma; RLUC: Renilla luciferase; RE: values and relative error; RSD: relative standard deviation; SI: selective index; SEM: standard error of the mean; SLAC: Shanghai Laboratory Animal Company; SPF: specific-pathogen-free; TC_50_: 50% toxicity concentration; TCID_50_: 50% tissue culture infective dose; T_max_: time at which the maximum concentration occurred; ULOQ: upper limit of quantitation; Vdss: volume of distribution at steady state; LD_50_: median lethal dose.

## Appendix A

**Table A1:** Inhibitory effects of ZSP1273 on influenza virus polymerase.

Compound	IC _50_ [34] *			
	1	2	3	Mean ± SEM **
ZSP1273	0.533	0.464	0.69	0.562 ± 0.116
VX-787	1.278	1.228	1.841	1.449 ± 0.34
Favipiravir	15,689	44,839	41,735	34,087.67 ± 16,009.21

* Note: IC_50_ values were calculated using the Reed–Muench method. The Reed–Muench formula is as follows: Proportionate distance = (% inhibition above 50% − 50%)/(% inhibition above 50% − % inhibition below 50%); IC_50_ = drug concentration with the %inhibition below 50% × 2^proportionate distance^. ** A total of three independent experiments were conducted to calculate the mean ± SEM in this assay.

**Table A2:** Summary of the IC_50_ of ZSP1273 against 24 kinases by kinase profiling.

Kinase	IC_50_ (nM) *		
	ZSP1273	VX-787	Staurosporine
ABL1	>10,000	>10,000	171
Aurora A	527	1115	4
CAMK2 alpha	>10,000	>10,000	1
CDK2	>10,000	>10,000	4
CHK1	>10,000	>10,000	6
CK1 alpha	>10,000	>10,000	>10,000
DYRK3	>10,000	>10,000	165
EPHA2	>10,000	>10,000	675
FGFR1	>10,000	>10,000	26
GSK3 beta	>10,000	>3333	60
MAP4K4	>10,000	1782	2
JAK2	>10,000	>10,000	6
JNK1	>10,000	>10,000	940
KDR	>10,000	>10,000	37
LCK	>10,000	>10,000	15
MAPKAPK2	>10,000	>10,000	318
MINK	>10,000	>3333	1
MST4	>10,000	>10,000	4
p38 alpha	>10,000	>10,000	>10,000
PDK1	>10,000	>10,000	5
PKA	>10,000	>10,000	12
SRC	>10,000	>10,000	42
TAOK2	>10,000	>10,000	18
TNIK	>10,000	>3333	13

* Note: Kinase activity was measured using Z’-LYTE^®^ technology (Invitrogen) based on the principle of fluorescence resonance energy transfer (FRET). The IC_50_ value was calculated by determining the % inhibition of kinase by the drug. ZSP1273, VX-787, and staurosporine were tested at maximum concentrations of 10,000 nM. The IC_50_ values of VX-787 against GSK3 beta, MINK, and TNIK were more than 3333 nM according to XLfit5 (formula 205).

**Table A3:** Agonistic effects of ZSP1273 against GPCR targets.

Target	ZSP1273		Positive Control	
	EC_50_ (nM) *	Activity at Maximum Concentration (%)	EC_50_ (nM)	Compound Name
op-delta	>12,000 *	−10.64	0.66	DPDPE
op-kappa	>12,000	−18.96	52.42	U69593
op-mu	>12,000	−3.45	1.07	DAMGO
D1	>12,000	−2.15	0.63	(±)-SKF-38393
D2	>12,000	−0.06	2.77	Dopamine
Alpha1a	>12,000	−2.93	8.29	(−)-Epinephrine (E4642)
Alpha2a	>12,000	−2.1	0.22	UK14304
M1	>12,000	−3.22	0.78	Arecaidine propargyl ester
M2	>12,000	−3	76.22	Arecaidine propargyl ester
M3	>12,000	−2.89	0.31	Arecaidine propargyl ester
Beta1	>12,000	0.54	3.24	(−)-Epinephrine
Beta2	>12,000	1.43	0.58	(−)-Epinephrine
AD2	>12,000	3.38	27.81	NECA
Eta	>12,000	−1.16	9.71	Endothelin 1
H1	>12,000	0.73	1.3	Histamine
H2	>12,000	−3.46	1344	Amthamine dihydrobromide
CCKa	>12,000	−4.4	71.39	CCK-4
5HT1A	>12,000	16.98	0.12	5-CT
5HT2A	>12,000	−21.27	9.35	5-CT
CB1	>12,000	−12.1	849.7	WIN55212-2
CB2	>12,000	−17.1	119.5	CP55940
V1a	>12,000	6.27	0.78	[Arg8]Vasopressin-acetate

* Note: ZSP1273 was tested at a maximum concentration of 12,000 nM. The agonistic effects of the test compounds against the targets were tested using FLIPR technology at a cellular level, and their safety was preliminarily evaluated. EC_50_ values were analyzed using the Prism statistical software.

**Table A4:** Antagonistic effects of ZSP1273 against GPCR targets.

Target	ZSP1273		Positive Control	
	IC_50_ (nM) *	Inhibition at Maximum Concentration (%)	IC_50_ (nM)	Compound Name
op-delta	>10,000	7.37	1.78	Naltrindole
op-kappa	>10,000	−27.8	1.6	nor-Binaltorphimine dihydrochloride
op-mu	>10,000	−24.47	116.8	CTOP
D1	>10,000	−16.73	1.3	R(+)-SCH-23390 hydrochloride
D2	>10,000	13.57	6.03	Amisulpride
Alpha1a	>10,000	−56.5	3.65	5-Methylurapidil
Alpha2a	>10,000	−9.3	26.07	Yohimbine hydrochloride
M1	>10,000	−0.95	65.02	Pirenzepine dihydrochloride
M2	>10,000	−55.66	0.09	Atropine
M3	>10,000	−4.78	1.27	Atropine
Beta1	>10,000	−22.66	3.12	Carvedilol
Beta2	>10,000	−33.19	5.35	BQ-123
AD2	>10,000	43.84	0.11	CGS-15943
Eta	>10,000	9.07	0.79	BQ-123
H1	>10,000	13.38	11.86	Pyrilamine maleate salt
H2	>10,000	42.39	280.9	Cimetidine
CCKa	>10,000	−17.56	255.5	Lorglumide
5HT1A	>10,000	19.04	92.8	Methiothepin mesylate salt
5HT2A	>10,000	2.56	71.67	Methiothepin mesylate salt
CB1	>10,000	−77.18	16.67	Rimonabant hydrochloride
CB2	>10,000	41.96	233.5	AM630
V1a	>10,000	7.24	39.99	[β-Mercapto-β,β-cyclopentamethylenepropionyl1, O-Et-Tyr2, Val4, Arg8]-Vasopressin

* Note: ZSP1273 was tested at a maximum concentration of 10,000 nM. The antagonistic effects of the test compounds against the targets were tested using FLIPR technology at a cellular level, and their safety was preliminarily evaluated. IC_50_ values were analyzed using the Prism statistical software.

**Table A5:** Inhibitory activity of ZSP1273 against influenza virus strains (H1N1 and H3N2).

Virus Strain/Cell	ZSP1273	VX-787	Baloxavir
	(nM)	(nM)	(nM)
Influenza virus A/California/07/2009 (H1N1)pdm09 *	0.02 ± 0.003	0.24 ± 0.14	1.06 ± 0.22
Influenza virus A/California/2/2014 (H3N2) *	0.01 ± 0.0002	0.15 ± 0.02	0.61 ± 0.02
MDCK (37 °C) **	1.21 ± 0.34	14.86 ± 2.47	14.36 ± 1.52

Note: * The data are presented as EC_50_ (mean ± SD) measured by the cytopathic effect method in MDCK cells, and the inhibitory activity of the compounds was analyzed by nonlinear fitting using GraphPad Prism (Version 5) software to calculate the EC_50_. ** Data are presented as CC_50_ (mean ± SD) measured by cytotoxicity experiments in MDCK cells, and the cell viability of the compounds was analyzed by nonlinear fitting using GraphPad Prism (Version 5) software to calculate the CC_50_.
